# Identification of Putative Precursor Genes for the Biosynthesis of Cannabinoid-Like Compound in *Radula marginata*

**DOI:** 10.3389/fpls.2018.00537

**Published:** 2018-05-09

**Authors:** Tajammul Hussain, Blue Plunkett, Mahwish Ejaz, Richard V. Espley, Oliver Kayser

**Affiliations:** ^1^Department of Technical Biochemistry, TU Dortmund University, Dortmund, Germany; ^2^The New Zealand Institute for Plant & Food Research Limited (PFR), Auckland, New Zealand; ^3^Max Planck Institute for Plant Breeding Research, Cologne, Germany

**Keywords:** *Radula marginata*, *de novo* transcriptomic assembly, biosynthetic metabolomics pathways, plant cannabinoids, *Radula*, a prospective alternate to cannabinoids

## Abstract

The liverwort *Radula marginata* belongs to the bryophyte division of land plants and is a prospective alternate source of cannabinoid-like compounds. However, mechanistic insights into the molecular pathways directing the synthesis of these cannabinoid-like compounds have been hindered due to the lack of genetic information. This prompted us to do deep sequencing, *de novo* assembly and annotation of *R. marginata* transcriptome, which resulted in the identification and validation of the genes for cannabinoid biosynthetic pathway. In total, we have identified 11,421 putative genes encoding 1,554 enzymes from 145 biosynthetic pathways. Interestingly, we have identified all the upstream genes of the central precursor of cannabinoid biosynthesis, cannabigerolic acid (CBGA), including its two first intermediates, stilbene acid (SA) and geranyl diphosphate (GPP). Expression of all these genes was validated using quantitative real-time PCR. We have characterized the protein structure of stilbene synthase (STS), which is considered as a homolog of olivetolic acid in *R. marginata*. Moreover, the metabolomics approach enabled us to identify CBGA-analogous compounds using electrospray ionization mass spectrometry (ESI-MS/MS) and gas chromatography mass spectrometry (GC-MS). Transcriptomic analysis revealed 1085 transcription factors (TF) from 39 families. Comparative analysis showed that six TF families have been uniquely predicted in *R. marginata*. In addition, the bioinformatics analysis predicted a large number of simple sequence repeats (SSRs) and non-coding RNAs (ncRNAs). Our results collectively provide mechanistic insights into the putative precursor genes for the biosynthesis of cannabinoid-like compounds and a novel transcriptomic resource for *R. marginata*. The large-scale transcriptomic resource generated in this study would further serve as a reference transcriptome to explore the *Radulaceae* family.

## Introduction

The liverwort *Radula marginata* belongs to the bryophyte division of land plants. Bryophytes are a distinctive group of an early-diverged lineage of non-vascular land plants comprising around 20,000 species. This division includes mosses, hornworts, and liverworts. Liverworts, or Marchantiophyta, are the most abundant phylum, comprising almost 6,000–8,000 species with high diversity in their ecology, morphology and genetic variation (Qiu et al., 2007; Rubinstein et al., 2010; Ruhfel et al., 2014). These non-vascular plants are very simple, with small flattened bodies with overlapping scales (Buck, 1986). Liverworts evolved (from algae) as the earliest bryophyte group and thus are considered ancestors of the mosses, hornworts, and land plants (embryophytes) (Willis and McElwain, 2002). Because of their distinct evolutionary position as a basal group for the colonization of land plants, bryophytes are ideal for exploring the evolution of plants, genetic networks, and developmental variation (Bowman et al., 2007; Sharma et al., 2014).

Among three classes of liverworts, the Jungermanniopsida encompasses 85% of known liverwort species. This is further subdivided into three subclasses and each subclass has three orders. The order Jungermanniales of the subclass Jungermanniidae consists of 13 suborders and 47 families (Stotler and Crandall-Stotler, 1977; Ruggiero et al., 2015). The *Radulaceae* family comprises the *Radula* genus, with 283 species. The most important *Radula* species are *R. complanta, R. demissa* (Renner et al., 2013)*, R. jonesii* (Bouman et al., 1998), *R. kojana, R. laxiramea, R. visianica, R. perrotteii*, and *R. marginata* (Losada-Lima et al., 2007). *Radula* species have been reported to be rich in habitual diversity and are found in almost all ecosystems such as trees, rocks and soils throughout the world, from Antarctica's coastal area to the northern hemisphere and from Australian semi-arid regions to the Amazon rainforest (Hallingbäck and Hodgetts, 2001).

Two decades ago, *Radula* species were shown to be rich in secondary metabolites, such as terpenoids and phenolics. For example, prenyl bibenzyls were identified in *R. perrottetti, R. complanta*, and *R. kojana* species (Asakawa et al., 1991; Toyota et al., 1994). These compounds have a distinct carbon backbone structures which acts as a marker to differentiate *Radula* species (Ludwiczuk and Asakawa, 2008). These compounds have suggested biological as well as pharmaceutical significance, having antifungal, antioxidant, antimicrobial and cytotoxic activities. Perrottetinene and its acid (perrottetinenic acid) have been identified in *R. marginata* (Toyota et al., 2002; Park and Lee, 2010). Interestingly, these compounds are structural analogs of tetrahydrocannabinol (Δ9-THC), a psychopharmacological compound in *Cannabis sativa L*.

Cannabinoids are plant secondary metabolites and belong to the class of terpenophenolics which are predominantly found in *C. sativa*. These compounds have proven to have a wide-ranging role in numerous clinical applications. These compounds accumulate in specialized glandular structures known as trichomes (Flemming et al., 2009; Happyana et al., 2013). Since cannabinoids have been recognized for their clinical value, research has also been carried out on plants other than *C. sativa* that contain cannabinoid-like compounds. This has led to the identification of cannabidiol-like (CBD-like) compounds in *Linum usitatissimum* (Styrczewska et al., 2012). Likewise, cannabigerol-like (CBG-like) compounds were found in the South African flowering plant *Helichrysum umbraculigerum* (Bohlmann and Hoffmann, 1979). In addition, sesquiterpenoid-like β-caryophyllene was discovered in *Piper nigrum* (Tisserand and Young, 2014). Despite some reports on the identification of cannabinoid-like compounds in plants, their complex plant architecture makes it challenging as an alternative source of cannabinoids. Although cannabinoids have been identified in different plant species, there has been no report on psychopharmacologically essential compounds except the THC-like metabolites found in *R. marginata*. Recently, the reported agonistic activity of THC-like natural products from *R. marginata* with the cannabinoid receptor 1 (CB1) further confirmed its significance (Russo, 2016; Gachet et al., 2017; Soethoudt et al., 2017). Therefore, *R. marginata* could be a suitable alternate source because of its relatively simple architecture and a diversity of natural habitats. Thus a genetic-level understanding of secondary metabolic pathways that lead to the synthesis of cannabinoid-like natural compounds would be desirable.

Since whole genome sequencing is resource-intensive, we performed a *de novo* approach to assemble the *R. marginata* transcriptome to establish a reference dataset. In addition to the transcriptome, micro-transcriptomic traits like transcription factors (TFs), simple sequence repeats (SSRs), and non-coding RNAs (ncRNAs) were also identified to understand the regulation of these genes. Interestingly, candidate genes for almost all the enzymes required for the conversion of primary metabolites into geranyl diphosphate (GPP) were identified. Expression of all the genes was confirmed by quantitative real time PCR (RT-qPCR). In addition, stilbene synthase (STS) was identified, structurally analyzed, functionally validated and considered as the first intermediate rather than olivetolic acid for the production of CBGA-like compound. This study was designed firstly to discover genes, identify biosynthetic pathways and the regulation of these genes to understand the biological processes involved. Secondly, being the first such gene expression profiling for this family, it would also serve as the reference transcriptome in future exploration of the *Radulaceae*. Furthermore, it would provide the link to study the transition from liverworts to higher plants during evolution.

## Results

### Illumina NGS sequencing and *de novo* transcriptome assembly

In the absence of a reference genome, *de novo* assembly of *R. marginata* was performed to obtain a comprehensive reference transcriptome and to identify novel genes for the biosynthesis of secondary metabolites, in particular, cannabinoid biosynthesis. For this purpose, we performed paired-end RNA sequencing to determine the distant connections between the transcripts. RNA-sequencing yielded ~30 million raw reads for each pair with an average length of 250 bp. After filtering the raw reads for low base quality, trimming and adapter removal (see section Materials and Methods for details), we selected 91% (28,231,052) reads for the assembly (Table 1, Supplementary Table 1). Raw reads were *in-silico* normalized and assembled using Trinity (version 2.2.0). The *de novo* assembly resulted a total of 1,580,612 transcripts, with a median contig size of 303 bp, and a maximum of 24,899 bp. Since *de novo* assembly also generates many isoforms of a gene, particularly at high coverage areas, we only selected the longest isoform per gene, resulting in 1,482,641 transcripts (Supplementary Table 2). In addition, clustering analysis resulted in 501,849 non-redundant transcripts of an average length of 484 bp and N50 of 459 bp. To perform the assembly assessment, raw reads were mapped back to the *de novo* assembled transcriptome using Bowtei2 (version 2.2.6) (Langmead and Salzberg, 2012). In total, 98% of raw reads successfully mapped back to the transcripts, of which 72% of the reads were mapped to both forward and reverse reads (Supplementary Table 3).

**Table 1:** Sequencing summary.

No of raw reads	30,981,418
No of filtered reads	28,231,052 (91%)
No of assembled bases	608,201,850
No of assembled transcripts	1,580,612
Read mapped	20,315,784 (72%)
Maximum length (bp)	23,874
Median contig length	303
Minimum length (bp)	278

To determine the potential of all the putative genes to translate into full-length protein, open reading frames (ORFs) of these putative genes were predicted using the TransDecoder software package (Haas et al., 2013). The homology of these genes was searched from the protein database UniProtKB/Swiss-Prot (http://www.uniprot.org/) followed by the identification of the protein domains by the Hidden Markov Model based approach using PFAM (http://pfam.xfam.org/). Results from both searches were incorporated with the longest ORFs. The ORFs showing homology to known proteins were selected. Based on the gene structure, we have predicted four types of ORFs: complete, three prime partial (3′p), five prime partial (5′p) and the internal ORFs. The complete ORFs were defined as the target genes which were transcribed with a start codon followed by five prime UTR, exon(s) and stop codon with three prime UTR (5′UTR+exon+3′UTR). The 3′p ORFs lacked a stop codon consisting of only 5′UTR and exon, while the start codon was absent in 5′p ORFs, only having exon and 3′UTR. In contrast, the internal ORFs were transcribed from the start to the end of the transcripts and did not contain start and stop codons. We have identified 2,880, 2,977, and 5,104 ORFs that belonged to complete, 3′p and 5′p ORF types, respectively. The highest number of the ORFs (76,499) belonged to the internal type. Moreover, for each type of ORF, the number of ORFs identified on the positive and negative strands was similar as shown in the percentages (Supplementary Table 4).

Finally, after the selection of the longest isoform, clustering of assembled transcripts, mapping of raw reads, quantification and retention of the longest ORF peptide candidates resulted in a total of 87,460 transcripts. This high quality unique transcriptomic dataset were named as unigenes. The length of unigenes ranged from 278 to 23,874 bp, with most of the unigenes (93%) having a length of up to 1 kb (Table 2). These unigenes were further used for the functional annotation and other downstream analysis. Furthermore, these unigenes were considered as the tentative consensus transcripts (Tct) and were assigned unique identifiers with the prefix *Rm-Tct* (*R. marginata* tentative consensus transcripts). The raw sequencing data have been submitted to NCBI under the BioProject No. PRJNA430820, BioSample No. 08378951 and Sequence Read Archive No SRR6489347.

**Table 2:** Assembly statistics.

	**Transcripts**	**Contigs**	**Unigenes**
Number	1,580,612	217,702	87,460
Length	881,230,701	77,717,062	36,765,277
N50 (bp)	604	314	324
Percent GC	54.25	55.7	55.75
Median length (bp)	311	308	316
Averager length (bp)	557	359	425
200–500 nt	1,262,140	209,643	81,695
500–1,000 nt	205,061	3,788	1,984
1,000–2,000 nt	49,077	1,699	1,356
2,000–5,000 nt	43,227	2,205	2,064
5,000–10,000 nt	17,345	331	326
>10,000 nt	49	35	35

### Functional annotation

#### BLAST functional analysis

**Figure 1:**
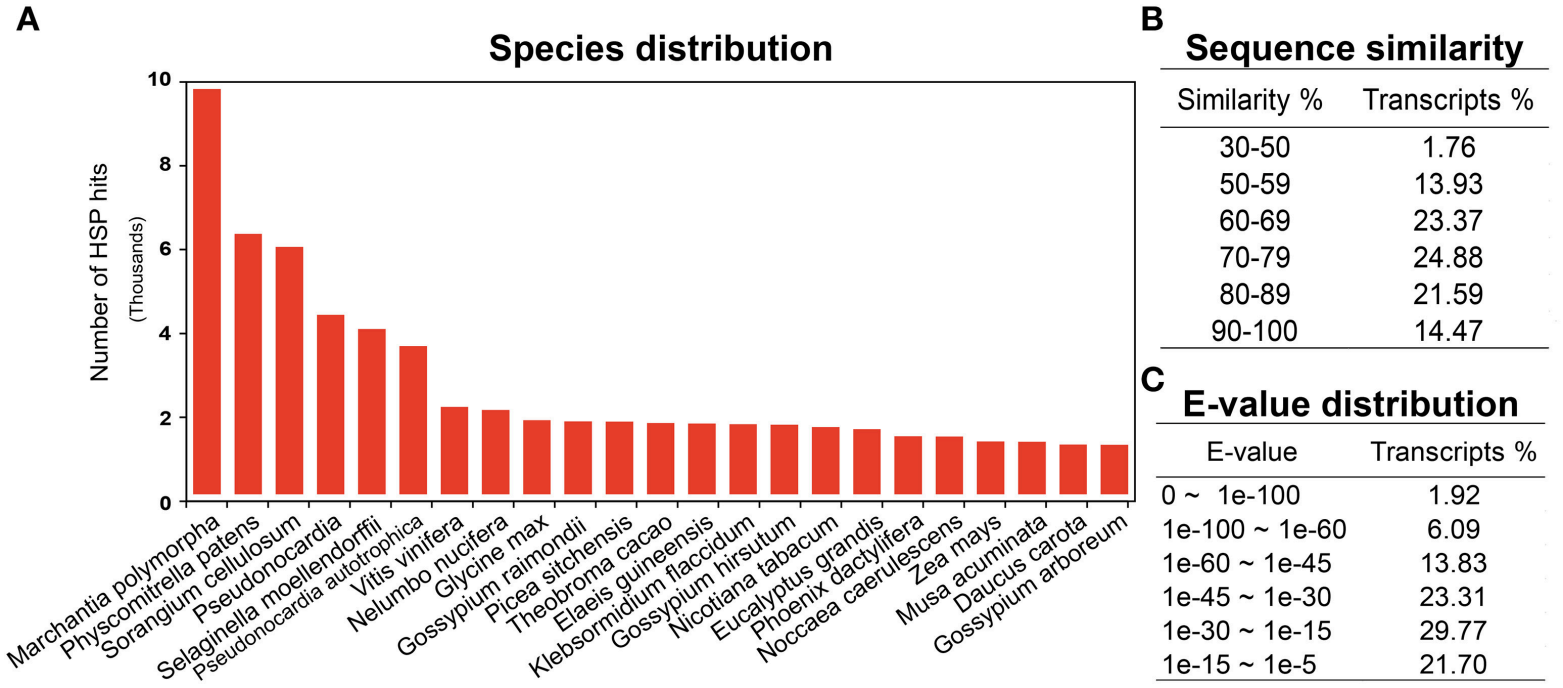
BLAST analysis. **(A)** Blast species distribution of closely related bryophyte species against NCBI non redundant (nr) database with an *E*-value of 1e-03. **(B)** Sequence similarity and **(C)** *E*-value distribution of BLAST results.

Annotation is one of the most critical steps for interpreting and precisely evaluating the transcriptomic assembly. For the preliminary functional analysis, homology of all the unigenes was searched using the blastx algorithm (Altschul et al., 1990) against the National Centre for Biotechnology Information (NCBI) https://www.ncbi.nlm.nih.gov/ non-redundant (nr) protein database. High quality identifiers for 68,715 (78%) unigenes were found with known protein coding potential with a cut-off *E*-value of 1e-05. The remaining 18,746 (21.5%) unigenes could not be matched with known protein entries in the database, because of lack of an existing genome sequence and EST information for *R. marginata*. Currently there are only 34 nucleotide and 13 protein entries available at NCBI. Percentage identity and *E*-values are two important parameters determining the quality of BLAST results. In our analysis, we found 98% of the blasted unigenes had sequence similarity between 50 and 90%, except 1.76% of the transcripts, which had a minimum sequence similarity of 31% (Figure 1B). Similarly, 23, 31, and 24% of unigenes had an *E*-value of up to 1e-15, 1e-30, and 1e-45, respectively. Only 2% of the sequences had an *E*-value equal to zero (Figure 1C). Higher percentage identity as well as a lower *E*-value of the majority of the blasted unigenes reflects the strength of the blast analysis. Furthermore, blast species distribution (Supplementary Data 1) showed that *Marchantia polymorphia* and *Physcomitrella patens* were the most abundant homologous species. Indeed, these two species are the closest relatives to *R. marginata*. The most represented species in blast hits are shown in Figure 1A.

#### InterProScan functional classification

**Figure 2:**
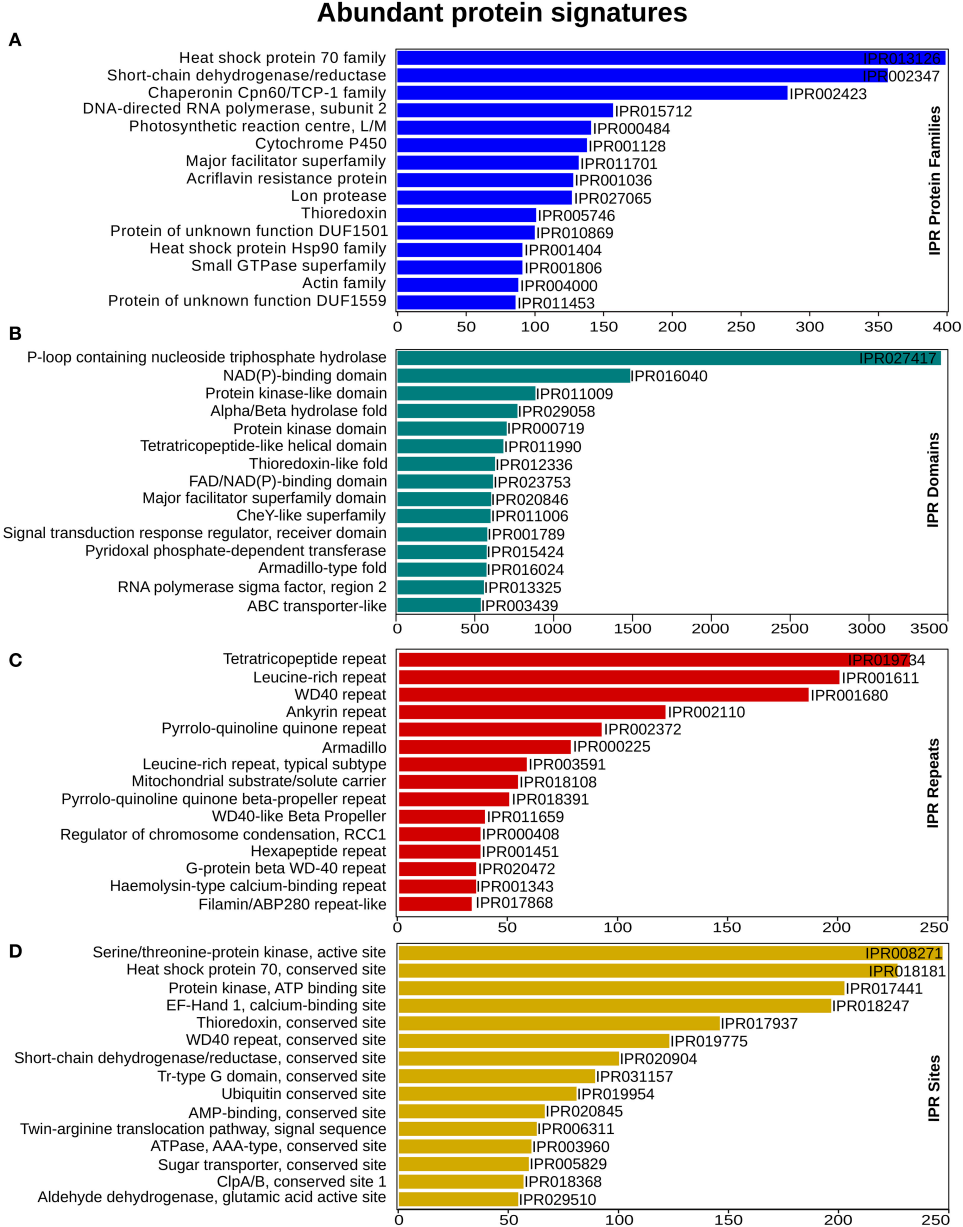
InterProScan functional analysis. **(A)** Annotation distribution of most significant protein families **(B)** domain identified **(C)** prominent repeats observed and **(D)** important sites determined. X-axis represents the number of genes associated with the respective protein signatures on Y-axis.

To enable a functional classification of unigenes, InterProScan (InterPro) https://www.ebi.ac.uk/interpro/ (release 60) was used. From this analysis, 65,415 (74%) unigenes matched with at least one protein signature. In total 3,487 protein family, 3,529 domains, 515 sites and 108 repeats had significant similarity, with 19,760, 60,021, 1,922, and 4,378 unigenes, respectively. “Heat shock protein 70 family” was the most abundant, with 399 unigenes, followed by the Short-chain dehydrogenase/reductase SDR and “Chaperonin Cpn60/TCP-1 family” that had 357 and 284 unigenes for each category (Figure 2A). Within the domain signatures, 2,935 unigenes had the “P-loop containing nucleoside triphosphate hydrolase” domain (Figure 2B). The Tetra-tri-co-peptide repeat, the Leucine-rich repeat and the WD40 repeat were the most recurrent that were found in 232, 200 and 186 transcripts, respectively (Figure 2C). Furthermore, out of the predicted site signatures, 68% (349) of the signatures were from the conserved category, followed by 19% (96) that belonged to the active sites. The serine/threonine-protein kinase active site was the most prevalent site present in 205 unigenes. The top most representative protein signatures for the four protein classification databases are shown in Figures 2A–D, Supplementary Figure 1A and Supplementary Data 2.

### Gene ontology classification

**Figure 3:**
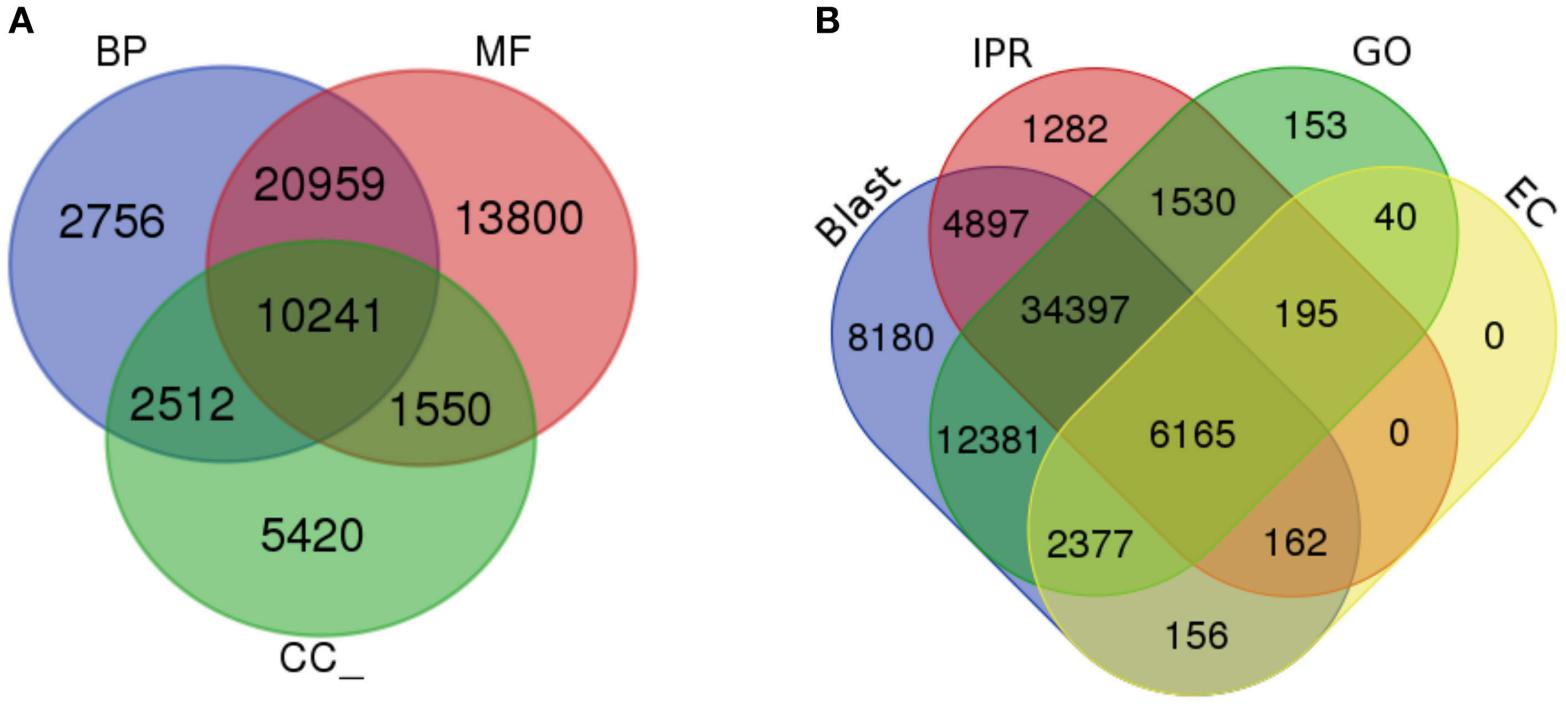
Gene Ontology analysis. **(A)** Gene ontology functional annotation for biological process (BP), Molecular function (MF), and Cellular component (CC). **(B)** Overall annotation results from BLAST, InterProScan (IPR), Gene ontology (GO), and KEGG pathway databases.

To determine the shared dynamic proteins among organisms and to understand the putative biological functions, gene ontology (GO) was analyzed (Ashburner et al., 2000). Based on the homology results from the NCBI nr database, GO terms were mapped to each unigene using a comprehensive annotation suite BLAST2GO (Conesa et al., 2005). Predicted InterPro scan protein signatures were also mapped for GO terms and finally an integrated annotation of unigenes was obtained after merging with blast-derived GO annotation. From the analysis, a total of 116,543 GOs were predicted from 4,820 functions. These GO terms were divided into three major categories: molecular function, biological process and cellular component. Fifty-one percent of the GOs (59,279) were predicted for the molecular function category (MF) followed by 34% (39,950) for biological processes (BP) and 15% (17,314) for cellular components (CC). Within the MF category, ATP binding, DNA binding, and oxidoreductase activity were significantly represented, with 5,359, 2,957, and 2,606 GOs for each type of function. Metabolic process (1,884) and regulation of transcription, DNA templated (1,744) were the most active BPs. CC were enriched with the membrane, membrane and cytoplasm integral components, with 4,080, 3,445, and 1,386 GOs. In total, 4,820 GO categories from 2,353 molecular functions, 1,849 biological processes and 618 cellular components were significantly present in the liverwort transcriptome. The total numbers of unigenes mapped with each GO category were 39,864, 31,268, and 14,369 for MF, BP, and CC, respectively. The ratio of GOs/unigenes was 1.49, with a minimum of one GO term per unigene to 27 GOs per unigenes (Figure 3A, Supplementary Figures 1B,C and Supplementary Data 3). From the complete annotation process we were able to generate 49,167 unigenes with functional annotations. In summary, 56% of the *de novo* assembled transcriptomic pool was annotated (Figure 3B).

The remaining 44% of the non-annotated were considered as unknown or orphan genes. Despite the lack of significant annotation, these unknown genes may have a possible functional potential as they were generated from the expressed transcripts (mRNA). They might either have potential binding sites for transcription factors (TFs), SSRs, or be ncRNAs.

### KEGG pathway network analysis

**Figure 4:**
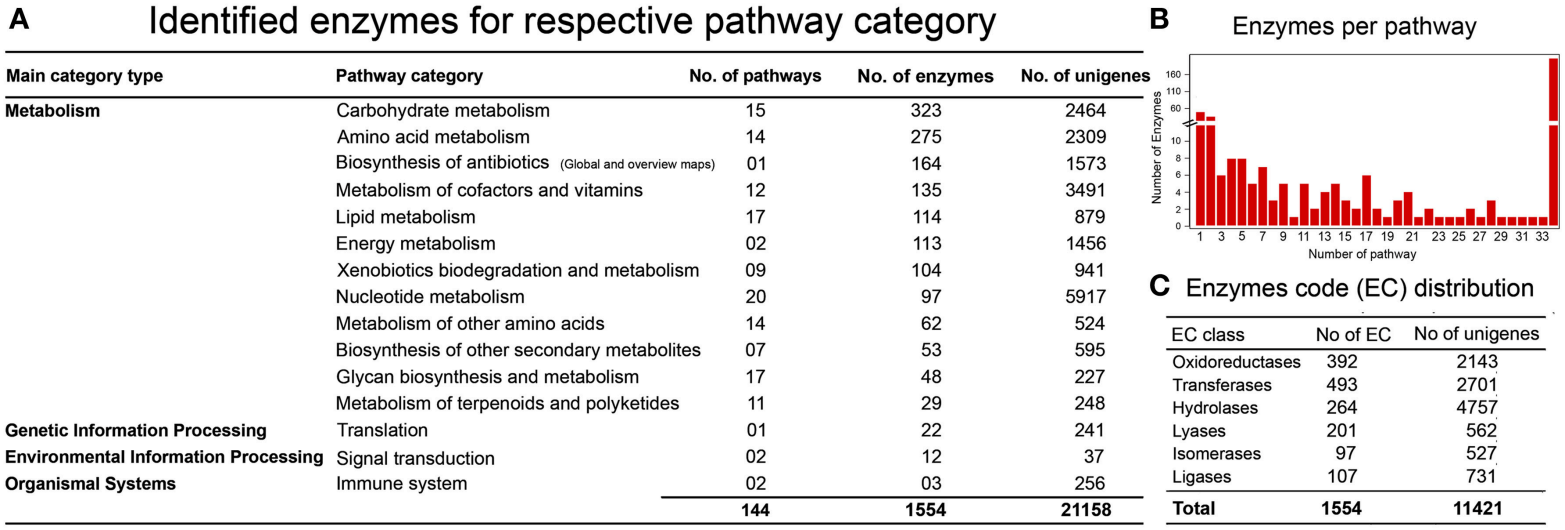
KEGG Pathway analysis. **(A)** Identification of genes encoding enzymes in 15 functional pathways among four main categories. **(B)** Distribution of enzymes per pathway, X-axis shows the number of pathways, while Y-axis represents the number of identified enzymes for each pathway. **(C)** Enzyme code distribution according to the enzyme class (EC) type.

One of the objectives in establishing a reference liverwort (*R. marginata*) transcriptome was to provide a resource for further NGS and proteomics studies. We further estimated the coverage of our newly assembled annotated reference transcriptome by determining the minimum coverage of general pathways involved in the MF and BP in *R. marginata*. To determine the rate of coverage, we analyzed more than 518 pathways listed in the Kyoto Encyclopedia of Genes and Genomes (KEGG) database. To identify active biological pathways, unigenes were scanned for the pathways based on analysis using KEGG (http://www.genome.jp/kegg/). As a result, 11,421 candidate genes were identified that encoded 1,554 enzymes in 145 biosynthesis pathways. These pathways belonged to 15 functional categories from “network of metabolism,” “environmental information processing,” “organismal processes” and “genetic information processing” (Figure 4A and Supplementary Data 4). Among the identified pathways, the carbohydrate metabolism pathway was mapped with the highest number of 323 enzymes. We identified 53 enzymes for secondary metabolite biosynthesis and 29 from the terpenoid metabolism and polyketide pathways (Supplementary Table 5). Moreover, the identified enzymes were broadly distributed into six major enzyme classifications (Figure 4B, Supplementary Table 6). Transferases were the most abundant enzymes, followed by the oxidoreductases and hydrolases. Distribution of the identified enzymes per pathways is shown in Figure 4C.

### Candidate genes for the biosynthesis of cannabinoid-like compounds in *R. marginata*

**Figure 5:**
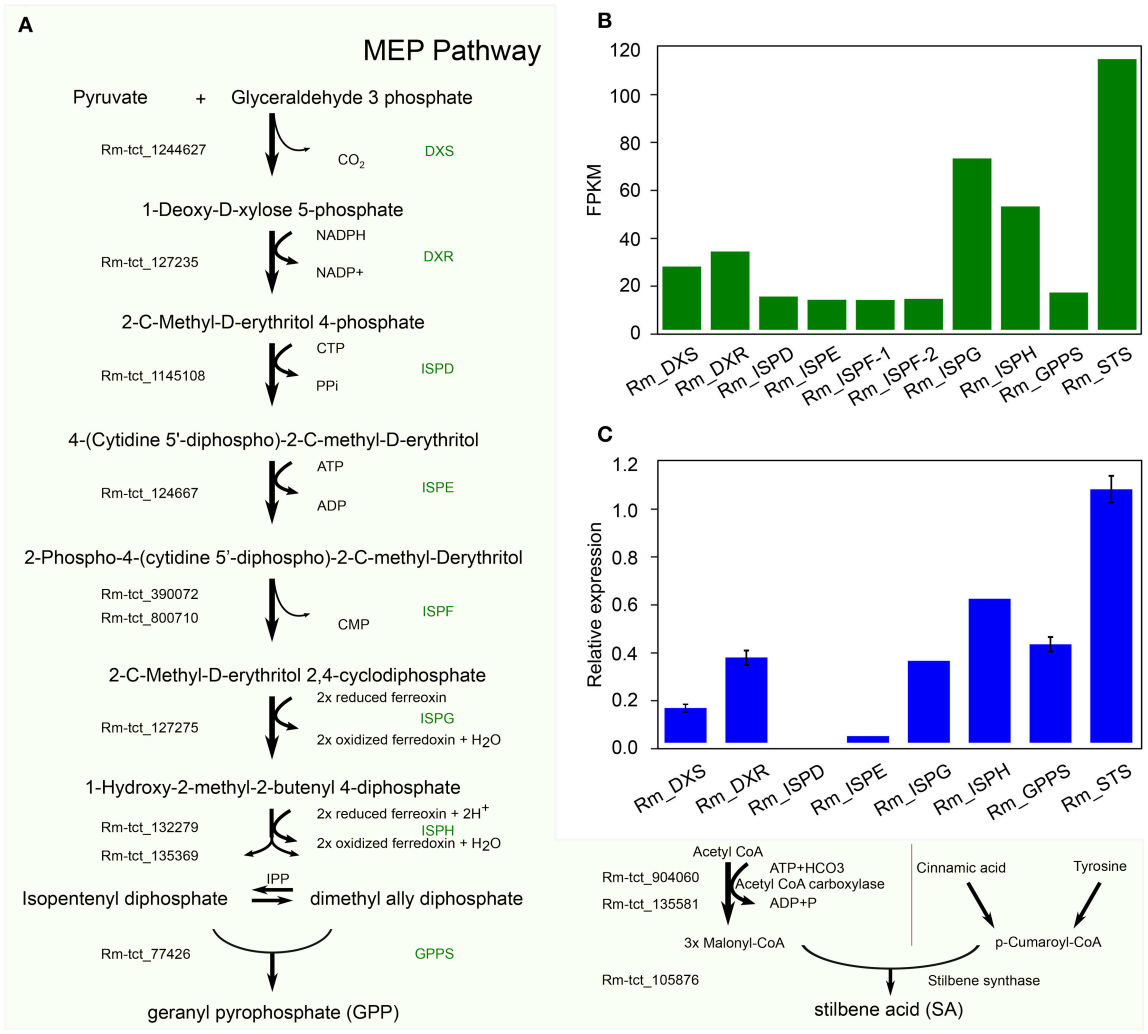
Candidate precursor genes for cannabinoid biosynthesis in *Radula marginata*. **(A)** Identification of candidate genes for cannabinoid biosynthesis in *R. marginata*. GPP transcripts as well as all the upstream genes were identified. Genes with Rm-tct_xxx prefix were identified in this study. **(B)** Transcriptomic expression of identified genes determined from FPKM. **(C)** Relative expression of candidate gene by real time quantitative PCR (RT-qPCR).

In *C. sativa* the biosynthesis of cannabinoids, such as tetrahydrocannabinolic acid (THCA), is the result of oxidative cyclization of cannabigerolic acid (CBGA) (Sirikantaramas et al., 2004; Taura et al., 2007; Stout et al., 2012; Andre et al., 2016; Zirpel et al., 2017). CBGA is the central precursor for cannabinoid biosynthesis, which is catalyzed by the alkylation of olivetolic acid (OA) with geranyl diphosphate (GPP). OA and GPP are derived from the polyketide and 2-C-Methyl-D-erthritol 4-phosphate (MEP) pathways, respectively. GPP is derived from a condensation reaction between two isoprene units, dimethylallyl pyrophosphate and isopentenyl pyrophosphate (Croteau and Purkett, 1989). Remarkably, we have identified all the potential homologous genes in *R. marginata* encoding the enzymes required for the conversions of pyruvate and D glyceraldehyde 3 phosphate into GPP, and also confirmed the expression through qRT-PCR (Figures 5A–C). The genes identified for GPP synthase had homology of 79.7% at the protein level with the GPP synthase in *Capsicum annuum*, and 75% with the large subunit of GPP synthase in *C. sativa*. In addition, we found that all the 15 potential binding sites of both isoprene units were conserved.

In contrast to OA in *C. sativa*, which is synthesized from the condensation of aliphatic CoA-tethered hexanoyl (hexanoyl-CoA), we found an analogous compound, stilbene acid (SA), in *R. marginata*. Compared with OA, SA catalyzes the condensation reaction from the CoA-tethered phenyl propanoid i.e., coumaroyl (P-coumaroyl-CoA). However, both require three molecules of malonyl-CoA by polyketide synthase (Fellermeier et al., 2001; Austin et al., 2004). Acetyl CoA carboxylase is the enzyme required for the carboxylation of acetyl-CoA to form malonyl-CoA, and has been identified in this study. Also, unlike the hexanoyl-acyl carrier protein, P-coumaroyl-CoA provides the “shuttle service” for the intermediate polyketide as well as substrates in the formation of SA by the enzymatic reaction of STS. The STS identified from *R. marginata* had 85% homology with chalcone synthase (CHS) and 84% homology with stilbencarboxylate synthase from *M. polymorph*. STS diverged from CHS as a result of gene duplication, from an evolutionary perspective (Tropf et al., 1994). Both catalyze the same reaction with different cyclization mechanisms, which lead to different products (Austin and Noel, 2003).

The CBGA analog was identified with the HPLC-ESI-MS-MS approach (Supplementary Figure 2A) and the metabolite with the same m/z was also observed from GC-MS analysis (Supplementary Figure 2B, Supplementary Data 5). The positive ESI mass spectrum of the CBGA bibenzyl analog showed an [M+H]+ ion at m/z 395, a predominant [M+H-H2O]+ ion at m/z 377, and fragment ions at m/z 271 [M+H-C9H16] and m/z 267 [M+H-H2O-C8H4]. The ion m/z 253 is probably the result of degradation processes eliminating two protons, resulting in [M+H-H2O-C9H16]. Together these indicate the presence of the CBGA analogue's bibenzyl structure in the methanolic extracts of *R. marginata*.

**Figure 6:**
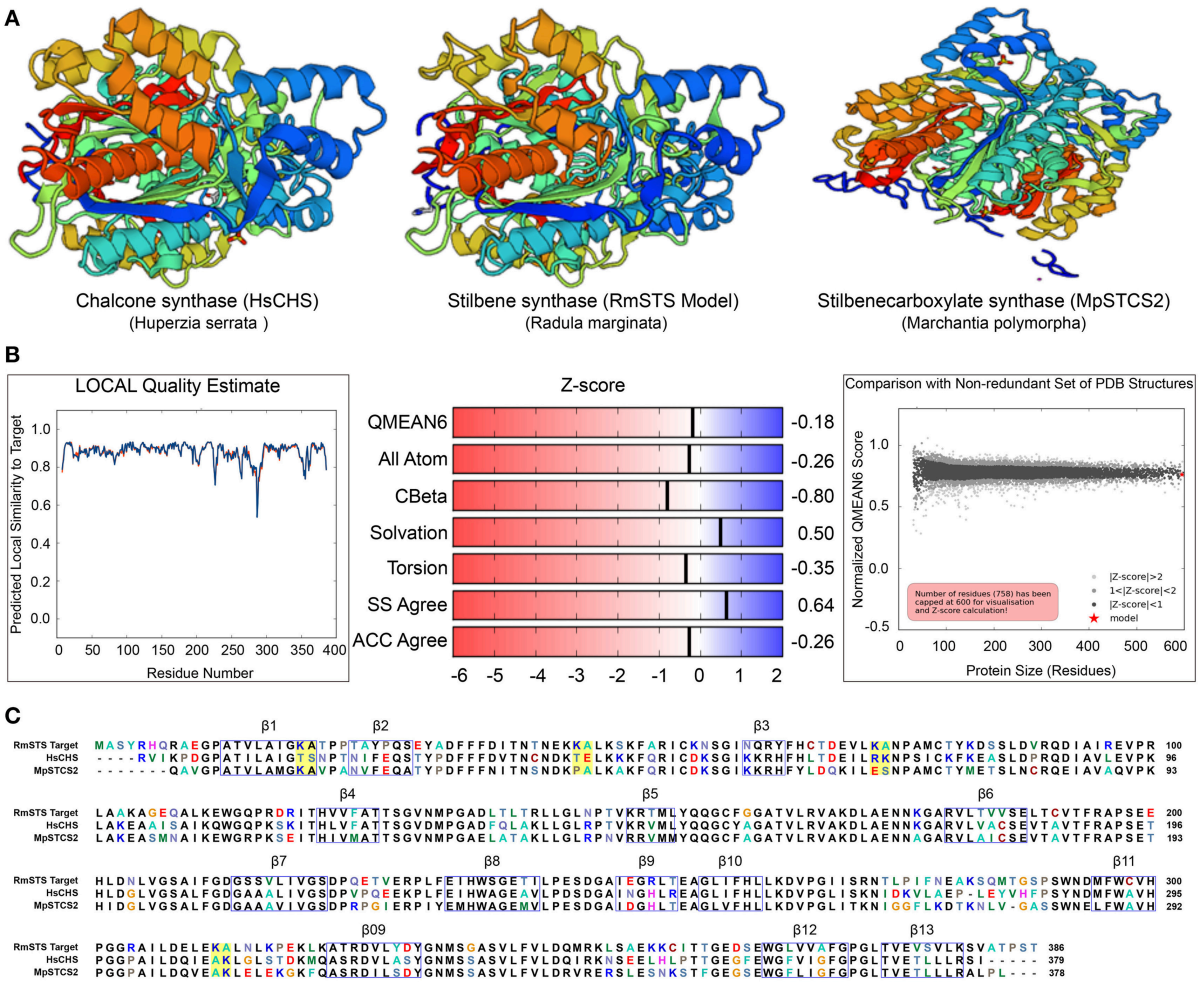
**(A)** Crystal structure of stilbene synthase model compared with Chalcone synthase- like polyketide (Left, 3awk.1) and Stilbene carboxylate synthase from *Marchantia polymorpha* (right, 2p0u.1. A). **(B)** Quality estimate of local similarity, Z score of the predicted STS model in *Radula marginata*. **(C)** Alignment of the identified stilbene synthase in *Radula marginata* with the respective template model, blue boxes are the beta sheets, yellow boxes in the alignment are the variable sites for both models used as well as in olivetolic acid from *Cannabis sativa*. Taken with permission from “https://swissmodel.expasy.org” (Arnold et al., 2006; Biasini et al., 2014).

To gain insights into the STS identified in *R. marginata*, we carried out structural analysis of the protein sequence. For this purpose, we used homology modeling to develop the structure of STS in comparison with the CHS-like PKS (PDB ID code 3awk) from *Huperzia serrata* and STS (PDB ID code 2p0u) from *Marchantia polymorpha*. Structural analysis revealed that the *R. marginata* STS shared 64 and 60% homology with these, respectively. In the model prediction, Lys-Ala was replaced with Thr-Ser, Thr-Glu, Arg-Lys, and Ala-Lys at positions 20–21, 46–47, 74–75, 312–313, respectively. Similarly, these variable sites except 20–21 were also found in STS from *M. polymorpha*, where they code Pro-Ala, Glu-Ser, and Ala-Lys amino acids. Likewise, OA in *C. sativa* also showed the same variable sites with the amino acid changes Thr-Ala, Thr-Gln, Ala-Gln, and Glu-Ala. Since STS diverged from CHS and OA was identified as the homolog of CHS, we speculate that these variable sites might have the potential for the diversification of the parental gene. However, the mechanism of the divergence and their catalytic activity of these potential sites need to be explored (Figure 6).

### Identification of transcription factors

**Figure 7:**
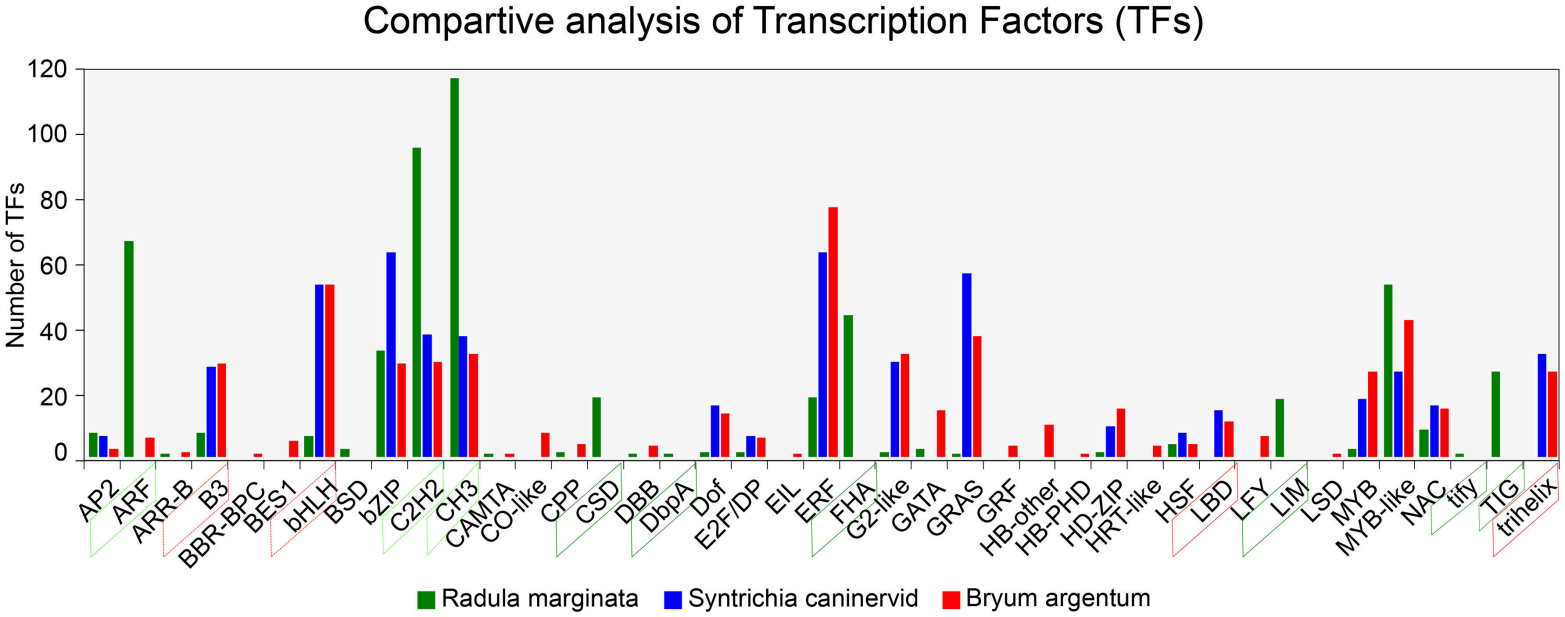
Comparative analysis of Transcription Factor families from *Radula marginata* and related moss species. TF families are on X-axis while number of TFs for each family shown on Y-axis. Green dotted box represents abundant TF families; those in red dotted box are less abundant, green solid-line box is for TF families that are exclusively observed in *R. marginata* compared with related moss species, while red solid-line box represents TF families that were not observed in *R. marginata*.

To understand gene regulation, unigenes were scanned using a Hidden Markov Models (HMM) (Finn et al., 2011)-based approach against the pfam (Finn et al., 2016) as well as the PlnTFDB (Pérez-Rodríguez et al., 2009) and PlantTFDB (Jin et al., 2014) databases. TFs were predicted according to the family assessment rule as described in PlantTFDB. To increase the accuracy, TFs with an *E*-value less than 1e-05 as well as an identity percentage < 50 were excluded from further analysis. After filtering we identified 1,085 TFs belonging to 39 TF families (Figure 7). We found that putative TF encoding regions were present in 3,449 unigenes. The total number of TFs identified in our study was higher than previously reported TFs in two desiccation-tolerant moss species, *Syntrichia caninervis* and *Bryum argenteum*, which contained 778 and 770 TFs, respectively. However, the number of TFs was less than in the model moss *P. patens*, which contains 1,156 TFs. Moreover, the 39 identified TF families in *R. marginata* were less than in the above-mentioned species, which contained 49, 50, and 53 TF families for each, respectively. Furthermore, the number of transcripts having a TF-encoding region was considerably higher than those in already-reported transcripts.

TF families are highly conserved in eukaryotic organisms, especially in plants. Despite the sequence conservation, the number of TFs for specific families varies among different species. This variation might be due to evolution or species specification. As for other plant species, evolutionary expansion/contraction was also observed in this study. For example, comparative analysis of three closely related moss species showed that the number of TFs within the ARF, C2H2, and CH3 families was higher in our data than in *B. argenteum*. In contrast, the B3 and bHLH families were relatively less abundant in the *R. marginata* transcriptome than in *S. caninervis* and *B. argenteum*. Moreover, six TF families—BSD, CSD, DbpA, FHA, LIM, tify, and TIG—were explicitly predicted in our data, confirming evolutionary expansion of TFs. On the other hand, we could not find any target sequences from B3 and trihelix TF families, which might have been due to evolutionary contraction. On this basis we might assume that the prediction of some TF families, as well as the apparent lack of other TF families, was due to evolutionary expansion and contraction, respectively.

The TF families: MYBs, Basic Leucine Zipper Domain (bZIP), AP2/ERF family proteins, NAC, Basic helix-loop-helix (bHLH), and DNA-binding One Zinc Finger (DOF), are considered those most closely involved in the regulation of secondary metabolism, development and growth in many plant species (Vom Endt et al., 2002; Li et al., 2015; Zhu et al., 2015). Interestingly, we found 156 TFs belonging to the above-mentioned families, with 66, 39, 9/22, 10, 8, and 2 TFs, respectively. Of these, MYB TFs were the most abundant, contributing up to 42% of the total TFs, some of which are proposed to regulate secondary metabolism by forming regulatory complexes with other TFs, such as bHLH and WD40 repeats in the regulation of phenylpropanoids (Espley et al., 2007; Liu et al., 2015). Moreover, the regulation of terpenoids has been associated with the APETALA2 (AP2), WRKY, and MYC families (Broun et al., 2006; Spyropoulou et al., 2014). In total, nine TFs from AP2 and three from WRKY were identified from our data. These predictive TFs might regulate aspects of secondary metabolism and terpenoid biosynthesis in *R. marginata* (Supplementary Table 7 and Supplementary Data 6).

### Simple sequence repeats (SSRs) identification

**Figure 8:**
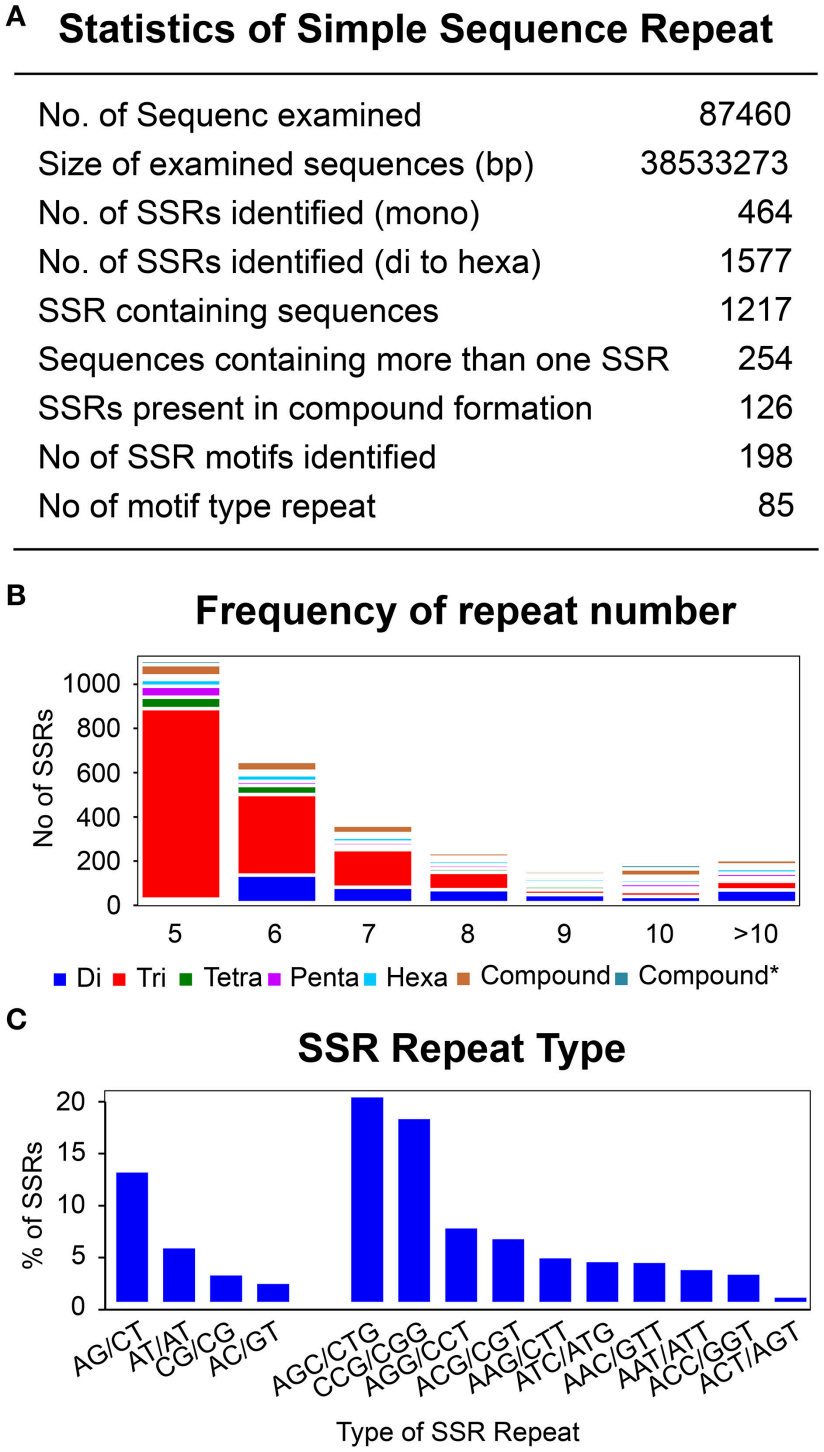
Identification of simple sequence repeats. **(A)** Statistic of simple sequence repeats. **(B)** Frequency of repeat number from 5 to 86 for di-, tri-, tetra-, Penta-, and hexa- type of SSR repeats. Y-axis represents the number of genes for each category of repeats type according to the repeat number on X-axis. **(C)** Most abundant di- and tri- repeats type distribution.

To identify SSRs, all the unigenes were screened using a microsatellite satellite identification tool (MISA) (http://pgrc.ipk-gatersleben.de/misa/). A total of 2,041 SSRs (mono to hexa-) were identified from 87,460 assembled transcripts (Figure 8A). Mononucleotide repeats can encounter a problem with a higher rate of homo-polymer errors associated with genotyping as well as sequencing (Gilles et al., 2011; Zhang and Huang, 2016). Hence, these were excluded from further SSR analysis. Of the remaining 1,577 di-nucleotide to hexa-nucleotide repeats, 90% of the SSRs belonged were di-nucleotides and tri-nucleotides. Tetra-, penta-, and hexa-nucleotide SSRs were found only in trace amounts, with 4, 3, and 3%, respectively (Figure 8B). The linkage potential of SSRs to loci was much higher with those SSRs identified from the annotated unigenes than for non-annotated (Li et al., 2014). The 1,577 SSRs were annotated with 1,217 unigenes with a minimum length of 12 bp. Moreover, 20% of the annotated unigenes (254) were found to have more than one SSR and 7% of the annotated SSRs (126) were found in compound formation. Based on sequence complementation, a total of 198 identified motifs were classified into 85 repeat types. Analysis showed that for di-nucleotide repeats AG/CT (12%) and AT/AT (5%) were the most abundant, while for trinucleotide repeats AGC/CTG (19%) as well as CCG/CGG (17%) showed the highest frequency. These specific repeats accounted for 53% of the total repeats found (Figure 8C, Supplementary Table 8 and Supplementary Data 7).

Repetition of the SSR motif varied from a minimum of five times up to 46 (86 for compound SSR). Although the repeat frequency was observed up to 86, the significance proportion of the motifs were below the repetition value of 10. Only 0.01% of the motifs were above 50, which was almost negligible as well as not being from the annotated SSRs. Similarly, a small fraction of motifs, 4.03%, were found to have a repetition of above 10. A significant proportion of the motifs lay within the repetition unit of 5 (48.54%), followed by the repetition of 6, 7, 8, and 9 with 26.46, 11.90, 5.65, and 1.46%, respectively. Furthermore, distribution of the repeat number within the repetition of 5 was decreased with the increase of motif length from tri to hexanucleotide. Trinucleotide motifs comprised the highest proportion, with 88%, followed by the tetra- (4%), penta- (3.4%), and hexa-nucleotides (1.4%) (La Rota et al., 2005; Hisano et al., 2008; Cloutier et al., 2009). This highest proportion of trinucleotide repeats was directly proportional to the accuracy. The possible reason for this is that expansion and contraction in tetra-, penta-, and hexa-nucleotide repeats may lead to a frame-shift mutation in the coding region (Metzgar et al., 2000; Morgante et al., 2002).

### Prediction of non-coding RNAs

To identify non-coding RNA genes (ncRNA), non-annotated unigenes were scanned for functional RNAs against Rfam database. Rfam http://rfam.xfam.org/ is a collection of RNA families and has three functional categories of non-coding RNA genes, structured *cis*-regulatory elements and self-splicing RNAs. ncRNA has been demonstrated to play roles in the regulation of gene expression at the post transcriptional level (Eddy and Hughes, 2001) as well as in maintaining genome stability by guiding RNA modification (Moazed, 2009). ncRNA genes produce a direct functional RNA molecule rather than being translated into proteins (Mattick and Mattick, 2010). From the analysis we predicted 2,043 ncRNA genes that belong to rRNA (1985), tRNA (46), sRNA (9), and snRNA (3) types (Supplementary Data 8). For the category of *cis-* regulation nine genes have significant homology. In order to determine the functionality of these predicted non coding genes further studies are required.

## Discussion

Next generation sequencing has become an essential tool for studying the genomic constitution of living organisms, especially those with high genome complexity. Despite the relatively low cost of sequencing, *de novo* assembly of whole genomes without prior sequence information is still costly and is computationally resource intensive. Compared with whole genome sequencing, *de novo* transcriptome analysis has made it possible to understand the genetic architecture of those organisms without a reference genome, at a low cost, and it is also computationally less intensive. Thus, a *de novo* transcriptome study is a valuable tool for identifying new genes, molecular markers, regulatory elements and the expression profile of genes (Verk et al., 2013). Indeed, this advancement has made it possible to study less well characterized species, for example, mosses and liverworts that have not been extensively studied compared with higher land plants such as rice (Zhang et al., 2010), poplar (Qiu et al., 2011) sesamum (Wei et al., 2011) as well as yeasts (Nagalakshmi et al., 2008) and animals (Feldmeyer et al., 2011). A *de novo* approach has previously been used for the moss species *S. caninervis* (Gao et al., 2014) and *B. argenteum* (Gao et al., 2015). Therefore, a *de novo* assembled transcriptome can be used to quantify the expression of genes and identify new genes.

*Radula marginata* (Liverworts), have been identified to contain a diverse array of secondary metabolites of high structural and medicinal properties for over the past two decades, such as perrottetinene and its acid (Toyota et al., 1994, 2002). However, there are only a few studies on liverwort species and most of those are related to biodiversity and molecular clock to infer the evolutionary relationships to land plants (Alaba et al., 2015; Singh et al., 2015; Honkanen et al., 2016). While *Radula* is an important plant order, it has not been investigated at the molecular and genomic level. Until now, only 34 nucleotide and 13 proteins have been reported for this liverwort species within the NCBI public database. Here, we report for the first time a *de novo* assembled transcriptome of *R. marginata*. In the RNA-seq analysis, we have identified all the upstream genes of the central precursor in cannabinoid biosynthesis. We have also validated the presence of CBGA-analogous by using HPLC ESI/MS-MS and GC-MS approach. We could identify all the prerequisite enzymes for the precursor which are likely to be conserved. However, we have identified stilbene synthase (SA) instead of olivetolic acid (OA), one of the CBGA precursor in *C. sativa*. Additionally, transcription factors (TFs), microsatellite markers (SSRs) and ncRNAs have also been identified. This *de novo* assembled reference transcriptomic dataset would provide a resource for exploring the *R. marginata*.

The sequence contents and quality of *de novo* assembled transcriptomes essentially depend on input material and further bioinformatics processing of the data. Assembly of the raw reads into contigs is the first step in the *de novo* transcriptomic study. We assembled 217,702 contigs from 30,981,418 raw reads. Because of splice site variation, assembly from Illumina sequencing generates many transcripts of different lengths for a single gene, where selection and identification of a single full-length transcript is a critical step in *de novo* assembly (Schliesky et al., 2012; Steijger et al., 2013). To remove this transcript redundancy (see section Materials and Methods) we finally obtained 87,460 unigenes. The total number of contigs and unigenes identified in our study was higher than the number of contigs (106,066) and unigenes (57,247) described in previously *de novo* assembled moss species (*B. argenteum*). This further illustrates the quality and accuracy of our assembly. Furthermore, the average length, GC contents and N50 value of these unigenes were comparable with the results from previous studies (Liang et al., 2013).

To estimate the quality of *de novo* assembly, raw reads were mapped back to the contigs generated from sequence assembly using bowtie2 with default parameters. Seventy-two percent of reads were successfully mapped back to the contigs and these mapping results are congruent with previous studies. However, the parameters used to check the quality of *de novo* assembly provide only rough estimates. Therefore, it is important to select all the transcripts that have coding potential and annotate all these transcripts to assign them a putative gene function. For this purpose, we used BLAST algorithms, such as blastn, blastx, and blastp with databases including NCBI non-redundant protein (nr), nucleotide (nt), Swiss-Prot (UniProtKB), cosmos (v1.6, *P*. patens proteins) and *M. polymorphia* genomes using a cut off value for homology search of at least 65% of identity and a stringent *E*-value of 1e-05. This allowed for 78% protein-coding transcripts with different degrees of homology.

Functional characterization of unigenes provides insights into the active BP, MF, and CC within an organism. To determine the functionality of genes, regarding regulation and expression, annotation is desirable. Therefore, all the unigenes were blasted with InterProScan (IPR), Gene ontology (GO), and Kyoto Encyclopedia of Genes and Genomes (KEGG) databases. In contrast to BLAST, 74% of the unigenes were mapped with the protein signature from 14 databases allied with IPR. Out of the total assembled unigenes scanned for GO terms, 56% of the unigenes had at least one GO which was more than the identified GO terms reported in the bryophyte moss species *S. caninervis* (48%) and in the non-model transcriptome of *Bacopa monnieri* (47%) (Jeena et al., 2017). However, *B. argenteum* and *P. patens* (Rensing et al., 2008; Zimmer et al., 2013) have 64 and 58% of GOs which is little higher than in our study. This might be due to an incomplete transcriptome or different cut-off values for transcriptome assembly processes.

To determine the interaction of functionally annotated unigenes of *R. marginata*, we performed KEGG pathway analysis (Kanehisa et al., 2006). This analysis elucidated the potential signal transduction biosynthesis pathways that were abundantly represented from our transcriptomic data. Out of 49,167 annotated unigenes, 11,421 were mapped with 145 signal transduction pathways which were greater than in other bryophyte species. For instance, *D. hirsute, S. caninervis*, and *B. argenteum* revealed the presence of 95, 119, and 127 pathways, respectively (Gao et al., 2014, 2015; Singh et al., 2015). Our results are also in line with those for other non-model organisms like *Sesamum indicum L*.,*Camelina sativa*, and *Callosobruchus maculatus* which showed 116, 119, and 119 pathways identified from their respective transcriptomes (Wei et al., 2011; Liang et al., 2013; Sayadi et al., 2016). The biosynthesis of the terpenoid backbone was the most significant pathway in the metabolism of terpenoids and polyketides with 84 unigenes identified that encoded 12 enzymes. Overall, network analysis revealed the presence of almost all the pathways involved in metabolism, of which carbohydrate metabolism was the most dominant with 323 enzymes followed by amino acid metabolism (275), metabolism of co-factors and vitamins (135) and lipid metabolism with 114 enzymes mapped to all of the pathways for each. However, we could not identify some of the secondary metabolic pathways, for example, zeatin, tetra cycline, and brassino steroid biosynthesis. It might be because the bryophyte transcriptomes have greater metabolic versatility than land plants which might have favored alternative metabolic pathways during evolution (Rensing et al., 2002; Oliver et al., 2004; Lang et al., 2005; Wood and Duff, 2009).

We have also identified the precursor genes for the synthesis of SA and GPP. Alkylation of OA with GPP results in the formation of CBGA which is the primary precursor for the cannabinoid biosynthesis in *C. sativa* (Fellermeier and Zenk, 1998). In contrast, we have identified SA in *R*. marginata and it is likely that SA acts in *R*. marginata as a first intermediate in place of OA to form CBGA-analogous. A metabolomics study also revealed the identification of the CBGA analogous and confirms our hypothesis that SA acts as the first intermediate where stilbene is the backbone of the identified compound. It is worth noting that the THCA like compound identified in *R. marginata* (Toyota et al., 2002) also has the stilbene backbone.

In our analysis, we were unable to annotate 44% of the transcriptome, and these were named as unknown genes. A similar percentage of unknown genes has also been observed in other moss species such as *S. caninervis* (41%), *B. argenteum* (36%) as well as other closely related liverwort species like *M. polymorpha* (43%) (Sharma et al., 2014). These high numbers of unknown genes might be due to the unavailability of sufficient genomic information for mosses in general and *R. marginata* specifically. To date, the only available reference genome for bryophytes is the moss species *P. patens* which also has 42% of unknown genes (Rensing et al., 2008; Ortiz-Ramírez et al., 2016). Therefore, we speculate that these unknown genes might play a role in gene regulation since they were generated from the expressed transcripts (mRNA). These unknown genes might be orphan genes and may have binding sites for TF, SSRs or ncRNAs.

The identified unigenes that have potential binding sites for 1088 TFs are consistent with those in other moss species such as *S. caninervis* with 778 and *B. argenteum* with 778 TFs, while *P. patens* has 1,156 TFs. However, the numbers of identified TF families are less than in the other related moss species. This variable number of TFs might be due to speciation events during evolution which explains the phenomenon of evolutionary expansion/contraction of TFs. We found six TF families that are unique to *R. marginata* which may be a result of expansion and three TF families that could not be detected from comparative analysis with related moss species. Although the number of transcription factors within a family varies, the sequence tends to remain conserved because of the conserved nature of TFs.

Our analysis of SSRs also revealed a large number of di- to hexa- repeats, of which 90% belongs to the di- and tri- repeat types. These data are correlated with *P. patens* where 91% of the SSRs comprised of di- and tri-nucleotide repeats. Some plant species such as sunflower, sesame, canola, *Arabidopsis*, peanut, sugar beet, cabbage, soybean, sweet potato, pea, and grape have a higher number of dinucleotide repeat motifs while trinucleotide type repeats are more frequent SSR motifs in cereals such as barley, rice, and wheat (Kumpatla and Mukhopadhyay, 2005; La Rota et al., 2005; Wei et al., 2011). Although repeat units were observed with a minimum of five to a maximum of 46 times, significant proportions of the repeat types were associated with five repeat units, as also observed in moss species.

## Conclusion

We describe the first high quality *de novo* assembled transcriptome, and have annotated the highest number of genes to date in *R. marginata*. Moreover, identification of the precursor genes in the cannabinoid biosynthesis pathway suggests that the cannabinoid pathway is likely to be conserved in lower and high land plants with the exception of first intermediate. These findings require further experimental work to confirm this proposed novelty. Overall, this study would serve as a new transcriptomic resource among the bryophyte species and also proposes *R. marginata* as an alternate to *C. sativa* for cannabinoid-like compounds.

## Materials and methods

### Liverworts collection

*Radula marginata* (liverworts) samples were collected from their basal habitat at Waitakere Ranges Regional Park, New Zealand. For metabolite extraction, material was transported in sealed plastic zip lock bags in liquid nitrogen to the Technical University Dortmund, Germany. For RNA extraction, samples were collected in liquid nitrogen and then stored at −80°C at The New Zealand Institute for Plant & Food Research Limited (PFR), Auckland, 1142, New Zealand.

### Extraction of RNAs

Total RNA was extracted from six samples using RNeasy™ Plant Mini Kit (Qiagen N.V., The Netherlands) according to the manufacturer's recommendations. To eliminate possible DNA contamination On-column DNaseI (Qiagen) digestion was added to the extraction protocol. RNA concentration was measured with Nanodrop ND-1000 spectrophotometer (NanoDrop Technologies, Wilmington, DE, USA) and quality was checked on agarose gel electrophoresis using Bioanalyzer agilant 2100 (Agilent Technologies, Santa Clara, CA, USA) was used to determine the integrity by RNA integrity number (RIN). Enrichment of mRNA by removing rRNA from total RNA was carried out by oligo (d) T beads (Qiagen, Hilden Germany). Purified mRNA was then transported in RNAStable plate (Biomatrica, San Diego, USA) to IGA Technology Services Udine, Italy.

### cDNA library preparation and illumina NGS sequencing

The total RNA from each of six samples was pooled and 2.5 μg of the pooled RNA was used to prepare cDNA according to the instructions of Illumina® (Illumina, San Diego, USA). The further steps in the preparation such as adapter insertion, interruption of the fragment, size selection and PCR amplification were performed at IGA Technology, Italy. Sixty million paired end reads of a length 250 bp were generated using Illumina HiSeq-2000 platform.

### Quality control

Illumina Hiseq-2000 sequencing platform (Illumina, San Diego, CA, USA) was used to sequence the transcriptome. FASTQC (version 0.11.24) (www.bioinformatics.babraham.ac.uk/projects/fastqc/) was used to determine the quality of the raw read. The base quality score was set to (Q20 = 1%) and all the reads below Q20 were considered as low quality (Andrews). Trimmomatics (version 0.36) was used to trim the low quality reads from 3′ and 5′ end using sliding window, leading and trailing to 4, 5, and 5 bases, respectively (Bolger et al., 2014). Adapter sequences present in the sequences were removed with cutadapt (version 1.9.1) (http://code.google.com/p/cutadapt/) (Martin, 2011). Read lengths with < 50 bp and reads with ambiguous “N” >5% were also dropped.

### Data processing and *de novo* transcriptomic assembly

The sequencing quality of raw reads was determined with FASTQC (https://www.bioinformatics.babraham.ac.uk/projects/fastqc/) software package. To avoid any false positive gene prediction adapters, low-quality base (higher than 20% nucleotides having a quality value ≤ 10) and reads (ambiguous nucleotide >5%) were removed with cutadapt and trimmomatic, respectively (Martin, 2011; Bolger et al., 2014). *In silico* read normalization was done before the assembly and then filtered normalized reads were *de novo* assembled using Trinity software package with default k-mer size of 25 (Haas et al., 2013). Inchworm, Chrysalis and Butterfly are the three independent modules for the assembly in Trinity. At the first step of assembly, Inchworm, raw reads are used to form a k-mer catalog followed by Chrysalis, using de Bruijn graph approach (based on distance and relation) to constructs the contigs. Finally, Butterfly connects all possible de Bruijn graph to construct the full-length transcripts as well as their splice variants as a result of alternative splicing.

To remove redundancy, assembled reads were clustered using CD-HIT-EST tool (Fu et al., 2012). To determine the protein coding region each transcript (putative gene) was *in silico* translated using TransDecoder (http://transdecoder.github.io). Among all the predicted frames, ORFs (open reading frames) peptide candidates were selected. Subsequently, the quality of the transcriptome was accessed by mapping raw reads back to the assembled reads using Bowtie2 (Langmead and Salzberg, 2012). Since *de novo* assembly is challenging for accurate mapping quantification, RNA-seq by Expectation-Maximization (RSEM) was used to determine the relative abundance of transcripts (Li and Dewey, 2011) which quantifies assembled transcripts with a high degree of accuracy by first preparing a reference set followed by the read mapping and abundance estimation. Afterwards, gene expression was calculated and reported as Transcripts per million (TPM) and fragment per kilobase of transcript per million mapped reads (FPKM) expected count numbers. Finally, the clustering of different isoforms of the transcripts (genes), selection of the longest putative ORF peptide candidate and the transcripts with at least TPM value > 1 were defined as a unigene.

### Functional annotation and characterization of unigenes

All the unigenes were searched against different public databases. BLASTx was used to determine the similarity against the NCBI non-redundant protein database (nr), Swiss-Prot, NCBI nucleotide database (nt) with stringent parameters (*E*-value 1e-05). For the functional characterization of unigenes, ORFs were scanned against InterProScan (IPR) release 60 (Finn et al., 2017). IPR suite scans unigenes against protein signatures (predictive models) from 14 affiliated databases resulting in a comprehensive protein annotation that includes protein superfamily classification, specific protein domains, repeats, and sites. Based on the homology results from nr databases, GO terms were mapped to each unigene using a comprehensive annotation suite BLAST2GO (Conesa et al., 2005). IPR protein annotation signatures were also mapped to GO terms and finally an integrated annotation was obtained after merging it with blast-derived GO annotation. Metabolic pathway analysis was done by similarity searching using blastx against the KEGG database (Kanehisa et al., 2004). The resulting unigenes and enzyme code were assigned and mapped to their respective pathway map.

### Identification of transcription factors (TFS)

Unigenes were scanned using Hidden Markov Model approach (HMM) in the HMMER software package against the Pfam, PlnTFDB, and PlantTFDB databases (Eddy, 2011; Finn et al., 2011, 2016). TFs were predicted according to the family assessment rule as described in PlantTFDB (Pérez-Rodríguez et al., 2009; Jin et al., 2014). The TFs retention criterion was set to an *E*-value less than 1e-05 and percentage identity of 50%.

### Identification of simple sequence repeats (SSRs)

To identify SSRs, we used microsatellite identification tool (MISA) (http://pgrc.ipk-gatersleben.de/misa/). All the unigenes were screened to identify potential sites for SSRs. Criteria were set such as minimum repeat unit number of 5 for di- to hexa- nucleotide repeats and a minimum repeat overlapping length of 100 bp. Mono nucleotide repeats were not included because of the higher error rate of homo polymer variation. Compound SSRs were also predicted with additional parameters as described above.

### Quantitative real-time PCR analysis

For quantitative real time PCR (qRT-PCR), the total RNA was extracted from four biological replicates of *R. marginata*. cDNA was synthesized according to the manufacturer's recommendations (QuantiTect® Reverse Transcription, QIAGEN Group). qRT-PCR assay was performed, using the LightCycler® SYBR Green I Master (Roche Diagnostics, Germany). Four technical replicates were used for each sample and quantified based on relative expression levels and normalized against the housekeeping gene *Actin* of *R. marginata*, using LightCycler® Software (Roche; version 1.5). Gene specific primers used for the qRT-PCR are listed in the Supplementary Table 9.

### HPLC ESI-MS/MS

One hundred milligrams of dried *R. marginata* material was grinded in 1 mL 80% methanol using a glass homogenizer. After centrifugation of the cell debris at 13,100 g at room temperature, the supernatant was analyzed by HPLC-ESI MS/MS analysis. Separation of the liverwort extract was performed by RP-HPLC (DAD) (Agilent 1260 Infinity HPLC, Waldbronn, Germany) equipped with a Poroshell 120 EC-C18, 2.1 × 100 mm, 2.7 μm column (Agilent, Waldbronn, Germany) with a flow rate of 0.7 mL min^−1^ at 40°C under isocratic conditions [50% (v/v) H_2_O with 0.1% (v/v) FA, 50% (v/v) ACN]. The identity of the liverwort cannabinoids was confirmed by mass and tandem mass spectra using a Bruker compact™ ESI-Q-TOF (Bruker, Bremen, Germany) operating at a positive ionization mode. Fragmentation was achieved at collision energy of 20.0 eV. The methodology was described in detail (Zirpel et al., 2017).

### GC-MS analysis

Fifty mg of the liquid nitrogen-ground *Radula* tissue was suspended in 100 μL of hexane, vortexed vigorously, and sonicated for 30 min. The samples were centrifuged at 16,000 g, for 10 min. The resulting supernatant was collected and analyzed. The GC/MS analysis was performed using TRACE 1310 gas chromatograph connected to a TSQ8000 triplequad MS (both Thermo Scientific). A DB-5 bonded-phase fused-silica capillary column (30 m length, 0.25 mm inner diameter, 0.25 μm film thickness) (J&W Scientific Co., USA) was used for separation. The GC oven temperature program was as follows: 2 min at 70°C, raised by 10°C/min to 300°C, and held for 10 min at 300°C. The total time of GC analysis was 36 min. Helium was used as the carrier gas at a flow rate of 1 mL/min. One microliter of each sample was injected in splitless mode. The initial injector temperature was 40°C for 0.1 min and after that time raised by 600°C/min to 350°C. The septum purge flow rate was 3 mL/min and the purge was turned on after 60 s. The transfer line and ion source temperatures were set to 250°C. Ion-source fragmentation was performed with 70 eV energy. Mass spectra were recorded in the mass range 35–650 m/z.

Data acquisition, automatic peak detection, mass spectrum deconvolution, retention index calculation, and library search were done by XCalibur and AMDIS software. The metabolites were automatically identified by library search (NIST library); the analyte was considered as identified when it passed a quality threshold: i.e., similarity index (SI) above 700 and matching retention index ± 10. The artifacts (alkanes, column bleed, plasticizers, MSTFA, and reagents) were identified analogously, and then excluded from further analysis. To obtain accurate peak areas for the deconvoluted components, unique quantification masses for each component were specified and the samples were reprocessed. The obtained profiles were normalized against the sum of chromatographic peak area (using the TIC approach).

### Structural analysis

A STS structural model was built with SWISS-MODEL by comparative approach using PDB ID code 3awk and 2p0u as a template (Arnold et al., 2006; Biasini et al., 2014).

## Author contributions

TH and OK: Wrote the first draft; TH, RE, and OK: Designed the experiment; TH and BP: Performed the experiments; TH and OK: Analyzed the data. TH, ME, RE, and OK: Wrote the manuscript. All authors contributed in the final version of the manuscript.

### Conflict of interest statement

The authors declare that the research was conducted in the absence of any commercial or financial relationships that could be construed as a potential conflict of interest.

## Supplementary material

Supplementary Table 1 Filtering summary of raw reads.

Supplementary Table 2 Isoform per gene summary.

Supplementary Table 3 Read mapping summary.

Supplementary Table 4 Predicted ORFs summary.

Supplementary Table 5 Enzymes identified related to secondary metabolites and “terpenoids and polykettides.”

Supplementary Table 6 Enzyme distribution according to subclass category.

Supplementary Table 7 Comparative summary of identified TFs.

Supplementary Table 8 Frequency of repeat number.

Supplementary Table 9 Oligonucleotide primers used for qPCR.

Supplementary Data 1 BLAST analysis summary.

Supplementary Data 2 IntrerProScan protein signature list.

Supplementary Data 3 List of gene ontologies (GO) identified from three categories.

Supplementary Data 4 List of identified enzymes associated with genes.

Supplementary Data 5 List of GC-MS detected compounds.

Supplementary Data 6 List of transcription factors (TFs).

Supplementary Data 7 List of simple sequence repeats (SSRs).

Supplementary Data 8 List of non-coding RNAs **(**ncRNAs).

Supplementary Figure 1 **(A)** Association of protein signatures for a specific transcript among PANTHER, GENE3D SMART, TIGERFAM, and SIGNALP protein databases. **(B)** Distribution of gene ontologies annotation from GO-levels 2 to 15 for each category of biological process (BP) molecular function (MF) and cellular components (CC). **(C)** Gene ontology distribution among three main categories of molecular function (MF), biological process (BP), and cellular components (CC). Y-axis represents the number of genes for their respective function/process/component as on X-axis.

Supplementary Figure 2 **(A)** Tandem mass spectra of the bibenzyl analog of cannabigerolic acid with m/z 395.214 **(B)** GC-MS chromatogram of *Radula marginata*.

## Abbreviations

BLAST: Basic local alignment search tool
GO: Gene ontology
BP: Biological Process
MF: Molecular Function
CC: Cellular Components
KEGG: Kyoto Encyclopedia of Genes and Genomes
NGS: Next Generation Sequencing
RT-qPCR: Real-time quantitative polymerase chain reaction
RPKM: Reads per kilobases per million reads
ncRNA: non-coding RNA
RSEM: RNA-seq by Expectation-Maximization
FPKM: Fragment per kilobase of transcript per million mapped reads
TPM: Transcripts per million
TFs: transcription factors
SSRs: simple sequence repeats
*Rm-Tct*: *Radula marginata* tentative consensus transcripts
SA: Stilbene acid
OA: Olivetolic acid
STS: Stilbene synthase
CHS: Chalcone synthase
PDB: Protein databank.
