# Supplementary material for: Identification of Putative Precursor Genes for the Biosynthesis of Cannabinoid-Like Compound in Radula marginata
Source: Front Plant Sci. 2018 May 9;9:537. doi: 10.3389/fpls.2018.00537 (PMC5954354; doi:10.3389/fpls.2018.00537)
Supplement: Supplementary Table 1 — Filtering summary of raw reads. [file Table_1.PDF]

Supplementary Table 1

Filtering summary of raw reads

	Q20	Q30
Phred score %	97.71	91.79
Percent GC	54.24	58.05
N50 (bp)	413	604

Supplementary Table 2

Isoform per gene summary

No of isoform	No of genes	Percent
1	1455425	98.16
2	13799	0.93
3	3956	0.27
4	2443	0.16
5	1317	0.09
6	1042	0.07
7	736	0.05
8	576	0.04
9	424	0.03
>10	2923	0.20
	**1482641**	**100**

Supplementary Table 3

Read mapping summary		
	Number	Percent
Both reads	20315784	71.96
Right only	461948	14.45
Left only	419104	13.11
Total reads	30981418	

Supplementary Table 4

Predicted ORFs summary

Type	strand	No of ORFs	total	%strand (+/-)
complete	+	1420	2880	49.31
	-	1460		50.69
3'partial	+	1438	2977	48.30
	-	1539		51.70
5'partial	+	2420	5104	47.41
	-	2684		52.59
Internal	+	37373	76499	48.85
	-	39126		51.15

Supplementary Table 5

Enzymes identified related to secondary metabolites and "terpenoids and polykettides"

KEGG Pathways	Pathway_ID	No. of genes	No. of enzymes	EC No.	Enzyme Name
**Biosynthesis of other secondary metabolites**					
Streptomycin biosynthesis	map00521	70	9	ec:2.6.1.5	transaminas
				ec:2.7.7.2	thymidylyltransferase
				ec:5.5.1.4	synthase
				ec:5.1.3.1	3,5epimerase
				ec:2.7.1.2	glucokinase(phosphorylating)
				ec:2.7.1.1	hexokinasetypeIVglucokinase
				ec:3.1.3.2	phosphatase
				ec:4.2.1.4	4,6dehydratase
				ec:1.1.1.1	2dehydrogenase
Tropane,piperidine and pyridine alkaloid biosynthesis	map00960	47	7	ec:2.6.1.5	transaminase
				ec:2.6.1.5	transaminase
				ec:2.6.1.9	transaminase
				ec:2.6.1.1	transaminase
				ec:3.1.1.1	tropineesterase
				ec:2.5.1.4	synthase
				ec:1.4.3.2	oxidase
Isoquinoline alkaloid biosynthesis	map00950	57	7	ec:1.4.3.2	oxidase
				ec:4.1.1.2	decarboxylase
				ec:2.6.1.5	transaminase
				ec:2.6.1.5	transaminase
				ec:2.6.1.1	transaminase
				ec:1.10.3.	oxidase
				ec:1.4.3.2	oxidase
Novo biocin biosynthesis	map00401	39	5	ec:2.6.1.5	transaminase
				ec:2.6.1.5	transaminase
				ec:2.6.1.9	transaminase
				ec:2.6.1.1	transaminase
				ec:1.3.1.1	dehydrogenase
Monobactam biosynthesis	map00261	38	4	ec:2.7.2.4	kinase
				ec:1.2.1.1	dehydrogenase
				ec:1.17.1.	reductase
				ec:2.7.7.4	adenylyltransferase
Penicillin and cephalosporin biosynthesis	map00311	11	4	ec:3.5.2.6	penicillinase
				ec:1.4.3.3	oxidase
				ec:3.5.1.1	amidase
				ec:5.1.1.1	epimerase
Caffeine metabolism	map00232	7	4	ec:1.17.3.	oxidase
				ec:1.7.3.3	uratehydroxylase
Acarbose and validamycin biosynthesis	map00525	14	2	ec:2.7.7.2	thymidylyltransferase
				ec:4.2.1.4	4,6dehydratase
Neomycin, kanamycin and gentamicin biosynthesis	map00524	19	2	ec:2.7.1.2	glucokinase(phosphorylating)
				ec:2.7.1.1	hexokinasetypeIVglucokinase
Indole alkaloid biosynthesis	map00901	3	2	ec:4.1.1.2	decarboxylase
				ec:4.3.3.2	synthase
Phenyl propanoid biosynthesis	map00940	234	2	ec:3.2.1.2	gentiobiase
				ec:1.11.1.	lactoperoxidase
Carbapenem biosynthesis	map00332	4	2	ec:1.2.1.4	dehydrogenase
				ec:2.7.2.1	5kinase
Flavonoid biosynthesis	map00941	1	1	ec:1.21.3.	synthase
Betalain biosynthesis	map00965	1	1	ec:4.1.1.2	decarboxylase
Phenazine biosynthesis	map00405	4	1	ec:4.1.3.2	synthase
Aflatoxin biosynthesis	map00254	43	1	ec:6.4.1.2	carboxylase
Glucosinolate biosynthesis	map00966	3	1	ec:2.6.1.4	transaminase
**Metabolism of terpenoids and polyketides**					
Terpenoid backbone biosynthesis	map00900	84	12	ec:2.7.4.2	kinase
				ec:4.1.1.3	decarboxylase
				ec:1.17.7.	synthase(ferredoxin)
				ec:2.2.1.7	synthase
				ec:2.7.1.1	5'-diphospho)-2-C-methyl-D-erythritolkinase
				ec:4.6.1.1	2-4-cyclodiphosphatesynthase
				ec:5.3.3.2	Delta-isomerase
				ec:2.3.1.9	C-acetyltransferase
				ec:2.3.3.1	synthase
				ec:1.1.1.2	reductoisomerase
				ec:2.1.1.1	O-methyltransferase
				ec:1.1.1.3	reductase(NADPH)
Geraniol degradation	map00281	69	4	ec:4.1.3.4	lyase
				ec:2.3.1.1	C-acyltransferase
				ec:4.2.1.1	hydratase
				ec:1.1.1.3	dehydrogenase
Polyketide sugar unit biosynthesis	map00523	16	3	ec:2.7.7.2	thymidylyltransferase
				ec:5.1.3.1	3-5-epimerase
				ec:4.2.1.4	4-6-dehydratase
Limonene and pinene degradation	map00903	21	2	ec:1.2.1.3	dehydrogenase(NAD+)
				ec:4.2.1.1	hydratase
Biosynthesis of siderophore group non ribosomal peptides	map01053	5	2	ec:1.3.1.2	dehydrogenase
				ec:5.4.4.2	synthase
Sesqui terpenoid and triterpenoid biosynthesis	map00909	1	1	ec:5.4.99.	cyclase
Biosynthesis of vancomycin group antibiotics	map01055	9	1	ec:4.2.1.4	4-6-dehydratase
Insecthormonebiosynthesis	map00981	7	1	ec:1.2.1.3	dehydrogenase(NAD+)
Carotenoid biosynthesis	map00906	1	1	ec:1.3.5.6	desaturase
Biosynthesis of 12-,14-and16-membered macrolides	map00522	4	1	ec:2.3.1.9	synthase
Biosynthesis of ansamycins	map01051	31	1	ec:2.2.1.1	glycolaldehydetransferase

Supplementary Table 6

Enzyme distribution according to subclass category

EC SubClasses	No. of genes		No. of enzyme	
**Oxidoreductases**				
1.1.- Acting on the CH-OH group of donors	434		137	
1.2.- Acting on the aldehyde or oxo group of donors	198		76	
1.3.- Acting on the CH-CH group of donors	153		29	
1.4.- Acting on the CH-NH(2) group of donors	127		44	
1.5.- Acting on the CH-NH group of donors	54		10	
1.6.- Acting on NADH or NADPH	182		5	
1.7.- Acting on other nitrogenous compounds as donors	21		8	
1.8.- Acting on a sulfur group of donors	144		13	
1.9.- Acting on a heme group of donors	57		1	
1.10.- Acting on diphenols and related substances as donors	32		5	
1.11.- Acting on a peroxide as acceptor	252		7	
1.12.- Acting on hydrogen as donors	30		2	
1.13.- Acting on single donors with incorporation of molecular oxygen (oxygenases). The oxygen incorporated need not be derived from O(2)	132		19	
1.14.- Acting on paired donors, with incorporation or reduction of molecular oxygen. The oxygen incorporated need not be derived from O(2)	163		16	
1.15.- Acting on superoxide as acceptor	45		0	
1.16.- Oxidizing metal ions	5		1	
1.17.- Acting on CH or CH(2) groups	94		14	
1.18.- Acting on iron-sulfur proteins as donors	14		4	
1.20.- Acting on phosphorus or arsenic in donors	1			
1.21.- Catalyzing the reaction X-H + Y-H = 'X-Y'	4		1	
1.97.- Other oxidoreductases	1			
		**2143**		**392**
**Transferases**				
2.1.- Transferring one-carbon groups	190		40	
2.2.- Transferring aldehyde or ketonic groups	101		16	
2.3.- Acyltransferases	284		82	
2.4.- Glycosyltransferases	250		51	
2.5.- Transferring alkyl or aryl groups, other than methyl groups	121		34	
2.6.- Transferring nitrogenous groups	69		77	
2.7.- Transferring phosphorus-containing groups	1591		188	
2.8.- Transferring sulfur-containing groups	94		4	
2.10.- Transferring molybdenum- or tungsten-containing groups	1		1	
		**2701**		**493**
**Hydrolases**				
3.1.- Acting on ester bonds	537		84	
3.2.- Glycosylases	167		50	
3.3.- Acting on ether bonds	49		4	
3.4.- Acting on peptide bonds (peptidases)	1149		3	
3.5.- Acting on carbon-nitrogen bonds, other than peptide bonds	224		83	
3.6.- Acting on acid anhydrides	2609		29	
3.7.- Acting on carbon-carbon bonds	11		3	
3.8.- Acting on halide bonds	6		6	
3.13.- Acting on carbon-sulfur bonds	5		2	
		**4757**		**264**
**Lyases**				
4.1.- Carbon-carbon lyases	229		80	
4.2.- Carbon-oxygen lyases	258		89	
4.3.- Carbon-nitrogen lyases	34		16	
4.4.- Carbon-sulfur lyases	19		11	
4.6.- Phosphorus-oxygen lyases	8		3	
4.99.- Other lyases	14		2	
		**562**		**201**
**Isomerases**				
5.1.- Racemases and epimerases	50		24	
5.2.- Cis-trans-isomerases	80		2	
5.3.- Intramolecular oxidoreductases	95		36	
5.4.- Intramolecular transferases	81		25	
5.5.- Intramolecular lyases	21		10	
5.99.- Other isomerases	200			
		**527**		**97**
**Ligases**				
6.1.- Forming carbon-oxygen bonds	223		21	
6.2.- Forming carbon-sulfur bonds	24		14	
6.3.- Forming carbon-nitrogen bonds	344		55	
6.4.- Forming carbon-carbon bonds	58		15	
6.5.- Forming phosphoric ester bonds	60		0	
6.6.- Forming nitrogen-metal bonds	22		2	
		**731**		**107**
		**11421**		**1554**

Supplementary Table 7

Comparative summary of identified TFs

TF family	No of TFs	Family members	TFs for member	Pfam	PlnTFDB	PlantTFDB
**AP2**	9	AP2	9	x	x	x
**ARR-B**	1	ARR-B	1	-	x	x
**ARF**	79	Arf	70	x	x	x
		ArfGap	8	x	x	x
		Arfaptin	1	x	x	x
**B3**	9	B3	5	x	x	x
		B3_4	4	x	x	x
**CPP**	2	CPP1-like	2	x	x	x
**C2H2**	113	zf-C2H2	56	x	x	x
		zf-C2H2_11	3	x	x	x
		zf-C2H2_3	1	x	x	x
		zf-C2H2_4	33	x	x	x
		zf-C2H2_6	9	x	x	x
		zf-C2H2_jaz	11	x	x	x
**C3H**	118	zf-C3HC4	34	x	x	x
		zf-C3HC4_2	50	x	x	x
		zf-C3HC4_3	28	x	x	x
		zf-C3HC4_4	6	x	x	x
**CAMTA**	1	CAMTA	1	-	x	x
**DBB**	1	DBB	1	-	x	x
**Dof**	2	zf-Dof	2	x	x	x
**E2F/DP**	2	E2F_TDP	2	x	x	x
**ERF**	22	eRF1_1	7	x	x	x
		eRF1_2	9	x	x	x
		eRF1_3	6	x	x	x
**G2-like**	2	G2-like	2	-	x	x
**GATA**	3	GATA	3	x	x	x
**GATase**	103	GATase	33	x	x	x
		GATase1_like	3	x	x	x
		GATase_2	22	x	x	x
		GATase_3	2	x	x	x
		GATase_4	7	x	x	x
		GATase_5	3	x	x	x
		GATase_6	17	x	x	x
		GATase_7	16	x	x	x
**GRAS**	1	GRAS	1	x	x	x
**HD-ZIP**	2	HD-ZIP_N	2	x	x	x
**HSF**	5	HSF_DNA-bind	5	x	x	x
**MYB**	3	Myb_Cef	3	x	x	x
**MYB_related**	63	Myb_DNA-binding	35	x	x	x
		Myb_DNA-bind_6	23	x	x	x
		Myb_DNA-bind_7	3	x	x	x
		Myb_CC_LHEQLE	2	x	x	x
**NAC**	10	NAC	10	x	x	x
**NFYA**	8	CBFB_NFYA	8	x	x	x
**NF-YB**	14	CBFD_NFYB_HMF	14	x	x	x
**SAP**	26	SAP	3	x	x	x
		SAPS	8	x	x	x
		SapB_1	9	x	x	x
		SapB_2	5	x	x	x
		SapC	1	x	x	x
**SBP**	5	SBP	3	x	x	x
		SBP56	2	x	x	x
**WRKY**	3	WRKY	3	x	x	x
**bHLH**	8	bHLH	8	-	x	x
**bZIP**	39	bZIP_1	19	x	x	x
		bZIP_2	17	x	x	x
		bZIP_C	1	x	x	x
		bZIP_Maf	2	x	x	x
**TCP**	7	Tcp11	7	x	x	x
**Cpn60_TCP1**	291	Cpn60_TCP1	291	x	x	x
**BSD**	3	BSD	3	x	x	-
**CSD**	22	CSD	22	x	x	-
**DbpA**	1	DbpA	1	x	x	-
**FHA**	52	FHA	52	x	x	-
**LIM**	21	LIM	18	x	x	-
		LIM_bind	3	x	x	-
**TIG**	31	TIG	31	x	x	-
**Tub**	2	Tub	2	x	x	-
**tify**	1	tify	1	x	x	-
	**1085**		**1085**			
**Others TFs**						
**SBP_bac**	454	SBP_bac_1	63	x	x	x
		SBP_bac_10	89	x	x	x
		SBP_bac_11	21	x	x	x
		SBP_bac_3	50	x	x	x
		SBP_bac_5	162	x	x	x
		SBP_bac_6	10	x	x	x
		SBP_bac_8	59	x	x	x
	454					

x =present, - = absent

Supplementary Table 8

Frequency of repeat number

Motif Length	Repeat unit number								
	5	6	7	8	9	10	>10	>50	Total
Di	-	108	53	42	20	12	41	-	**276**
Tri	845	349	154	62	5	6	23	-	**1444**
Tetra	36	24	-	1	1	-	-	-	62
Penta	33	3	1	1	-	3	4	-	45
Hexa	14	13	7	2	1	1	5	-	43
Compound	35	28	21	4	2	17	7		114
Compound*	2	-	-	-	-	3	-	-	5
Total	**965**	**525**	236	112	29	42	80	0	1989

Supplementary Table 9

Oligonucleotide primers used for qPCR

Primers	Gene	Sequence (5'-3')
Blue 147	Rma_DXS-F	ATTTTTCCGACAAGCCTCCA
Blue 148	Rma_DXS-R	CCGTAAAGATGGTCTCGGAC
Blue 123	Rma_DXR-F	CGAGTCTGCCGGTAAAATCT
Blue 124	Rma_DXR-R	ACTGTAGCAGCCATAGAAGC
Blue 125	Rma_ISPE-F	TCAGTTTGGGGGATGTCATC
Blue 126	Rma_ISPE-R	GATACAGGTTGAGGGCCTTG
Blue 149	Rma_ISPG-F	TCGCAGGTACTTTGACTTCC
Blue 150	Rma_ISPG-R	TGCTCCAACGAAACAGACAT
Blue 151	Rma_ISPH-F	CACGCCAGAAGGGAAAGATT
Blue 152	Rma_ISPH-R	GTGGTATCGACGATTTGCAC
Blue 115	Rma_GPPS-F	GGCCTTCTGTAACCCCAAAA
Blue 116	Rma_GPPS-R	CTGTTCCAGGTTGTCGATGA
Blue 117	Rma_STS-F	ACCTCACTCTGACGAGACTG
Blue 118	Rma_STS-R	CCTTGTTGTTCTCTGCGAGA
