# Supplementary material for: Identification of Putative Precursor Genes for the Biosynthesis of Cannabinoid-Like Compound in Radula marginata
Source: Front Plant Sci. 2018 May 9;9:537. doi: 10.3389/fpls.2018.00537 (PMC5954354; doi:10.3389/fpls.2018.00537)
Supplement: Supplementary Figure 1 — (A) Association of protein signatures for a specific transcript among PANTHER, GENE3D SMART, TIGERFAM, and SIGNALP protein databases. (B) Distribution of gene ontologies annotation from GO-levels 2 to 15 for each category of biological process (BP) molecular function (MF) and cellular components (CC). (C) Gene ontology distribution among three main categories of molecular function (MF), biological process (BP), and cellular components (CC). Y-axis represents the number of genes for their respective function/process/component as on X-axis. [file Image_1.PDF]

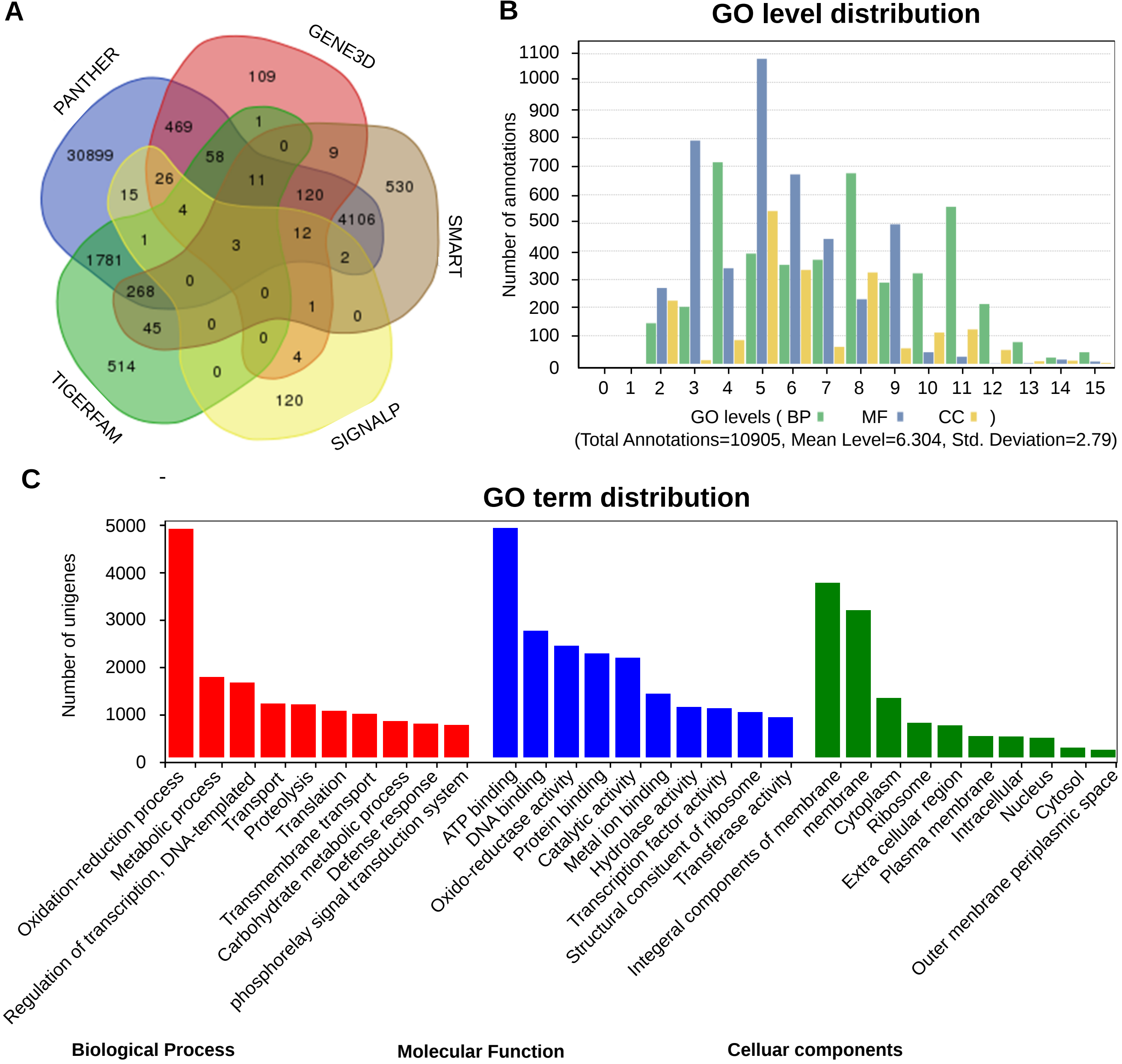

Supplementary Figure 1 A) Association of protein signatures for a specific transcript among PANTHER, GENE3D SMART, TIGERFAM and SIGNALP protein databases. **B)** Distribution of gene ontologies annotation from GO-levels 2 to 15 for each category of biological process (BP) molecular function (MF) and cellular components (CC). **C)** Gene ontology distribution among three main categories of molecular function (MF), biological process (BP), and cellular components (CC). Y-axis represents the number of genes for their respective function/process/component as on X-axis.
