# Supplementary figures and images for: Identification of Putative Precursor Genes for the Biosynthesis of Cannabinoid-Like Compound in Radula marginata
Source: Front Plant Sci. 2018 May 9;9:537. doi: 10.3389/fpls.2018.00537 (PMC5954354; doi:10.3389/fpls.2018.00537)

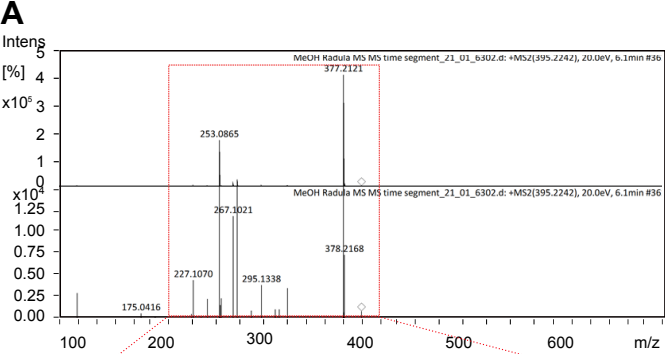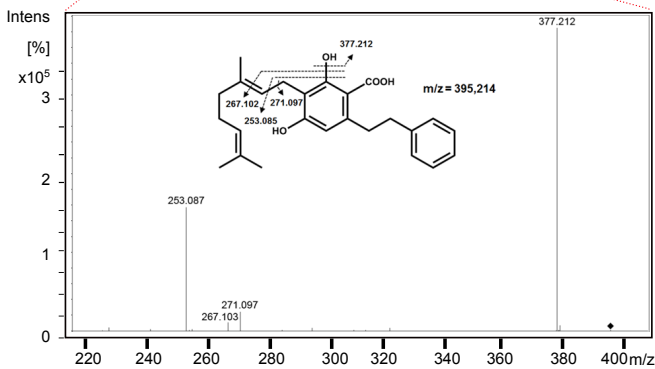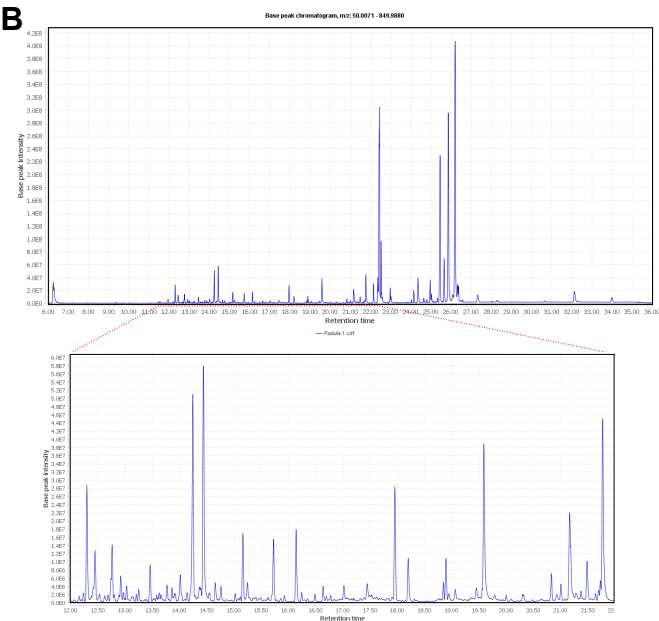

Supplement: Supplementary Figure 2 — (A) Tandem mass spectra of the bibenzyl analog of cannabigerolic acid with m/z 395.214 (B) GC-MS chromatogram of Radula marginata. [file Image_2.pdf]
